# Biofeedback in Breathing Pattern Training: A Systematic Review

**DOI:** 10.1155/carj/5567144

**Published:** 2026-11-15

**Authors:** Kristopher Bahadur, Julie Backley, Jake Bowd, John Dickinson

**Affiliations:** ^1^ Sport, Exercise, and Rehabilitation Sciences University of Kent Chipperfield Building Canterbury CT2 7PE Kent UK kent.ac.uk; ^2^ Institute of Sport, Exercise and Health 170 Tottenham Court Road London W1T 7HA UK

**Keywords:** biofeedback, breathing pattern disorders, breathing pattern training, dysfunctional breathing patterns, systematic review

## Abstract

**Background:**

Breathing pattern training is commonly used in the management of breathing pattern disorders and has been associated with improvements in symptoms and quality of life. The integration of biofeedback, which provides real‐time physiological information, may enhance breathing pattern training. However, its added value remains unclear. This systematic review aims to synthesise the evidence for the use of biofeedback in breathing pattern training.

**Methods:**

This review followed the 2020 Preferred Reporting Items for Systematic Reviews and Meta‐Analyses guidelines. Electronic searches of twelve databases, registries and archives (APA PsycInfo; SPORTDiscus; CINAHL Plus with full text; Cochrane Library; PEDRo; PubMed; Scopus; Web of Science; ClinicalTrials.gov; International Clinical Trials Registry Platform; BioRxiv; MedRxiv) were performed. Risk of bias was assessed using Cochrane tools for randomised and nonrandomised studies. Quality of evidence was evaluated using Grading of Recommendations Assessment, Development and Evaluation criteria.

**Results:**

Of 1196 records identified, 794 were screened and 103 were assessed for eligibility. Eight studies met the inclusion criteria. Whilst improvements in breathing pattern, symptom scores, respiratory measures and quality of life were highlighted, the evidence supporting this was based on few studies and in limited populations. Furthermore, substantial study heterogeneity, high risk of bias, and low levels of certainty and quality were highlighted. Additionally, improvements were more frequently observed within‐group than between‐group.

**Conclusions:**

Overall, there is a lack of reliable evidence of sufficient quality to suggest that the addition of biofeedback to breathing pattern training provides greater benefits than conventional training or exercise alone. Further high‐quality research is required to determine its effectiveness.

## 1. Introduction

The term breathing pattern disorder (BrPD) is used to describe a variety of complex and dysfunctional breathing patterns. The prevalence of BrPD is significant and are reported to affect approximately 9.5% of the general population in the United Kingdom [1]. Prevalence is reported to be 29%–34% in asthma cohorts [2, 3], rising to 64% in difficult‐to‐treat asthma [4], 50% in chronic obstructive pulmonary disease (COPD) populations [5], 22%–42% in elite athletes [6, 7] and 21% in children/teenagers [8]. BrPD is also common in anxiety‐related disorders [9–11], but suggestive figures from the literature are difficult to determine and are therefore not outlined here. More recently, BrPD has been identified in around 29% of patients with long COVID [12] and in about 32% of patients with new onset dysfunctional breathing after SARS‐CoV‐2 infection [13].

BrPD is multifaceted, multidimensional and complex [14–16], giving rise to a variety of symptoms, including breathlessness, hyperventilation, wheeze, stridor, chest pain, throat clearing, air hunger and dizziness [17–20]. Both physiological and psychological influences can contribute to the presentation of BrPD [15, 17, 21]. Health‐related quality of life (QOL) measures are described to be reduced in individuals with BrPD [17, 22, 23], further highlighting significant impact.

Breathing pattern training (BrPT) is one of the interventions used in the management of BrPD [24] and has been suggested to be the first‐line approach in physiotherapy guidelines [25]. BrPT has been associated with improvements in symptoms [26–28] and in measures such as Nijmegen scores [28, 29] and breath‐hold times [27]. Improvements in asthma outcomes [29], QOL impact scores [26–29] and healthcare utilisation [23] have also been reported. Other strategies available within the BrPT toolbox may also include the incorporation of education and psychological support [14, 30], postural assessment and correction [14, 17], diaphragmatic re‐education [18] and interventions aimed at improving thoracic and ribcage mobility [15]. Additionally, whilst the effects of inspiratory muscle training during BrPT have not been explicitly documented, improvements in chest wall kinematics have been outlined [31] and may provide opportunity for future interventions.

Biofeedback utilises technological devices to provide real‐time information on physiological parameters, enabling individuals to intentionally change the measured parameter to improve health and performance [32–34] and motivate behaviour change [35]. Biofeedback has been used within health [36–42], sport [43–49] and in a variety of different settings and populations, but its use as a component of BrPT is less well documented. An evidence map published in 2019 on the efficacy of biofeedback across medical conditions did not identify any high‐quality studies investigating its use in BrPT [33]. However, more recently, disordered breathing patterns have been acutely improved with real‐time optoelectronic plethysmography (OEP) phase angle feedback [50]. The phase angle describes the temporal movement of one chest wall compartment with respect to another [51]. A phase angle of 0 degrees represents synchronous compartmental movement, with increasing absolute phase angle values indicating greater chest wall compartmental asynchrony [52, 53].

A two‐round Delphi process with forty‐six international experts has recently been conducted to identify the optimal treatment for BrPD in adults [30]. Whilst this review identified BrPT with or without biofeedback to be essential in the management of BrPD, the evidence for the use of biofeedback was not evaluated or commented on. Additionally, no consensus on what this subcomponent of BrPT should look like was presented. Currently, it is unclear what the additional benefits of using biofeedback with BrPT are. The incorporation of biofeedback into BrPT may be beneficial if it results in greater improvements in breathing pattern. Therefore, the aim of this review was to investigate the potential benefit of including biofeedback in BrPT.

## 2. Methods

This systematic review is reported in line with the Preferred Reporting Items for Systematic Reviews and Meta‐Analyses (PRISMA) updated guidelines 2020 [54]. The November update consisted of a minor change in study title and an update of review status to completed. A Population, Intervention, Comparator and Outcome (PICO) framework was used to guide the systematic review. Please see Supporting Information 1.

### 2.1. Eligibility Criteria

No restrictions were placed on study design, publication date, language, type of setting or study duration. Studies investigating BrPT specifically for stress and anxiety management were excluded, as for the purpose of this study, it was considered to be a distinct separate group. Paced breathing where participants followed prompts, for example, using a metronome, was not considered to be biofeedback and not included in this study. Studies were not included if they were books, book chapters, conference abstracts/posters, standalone study protocols or single case studies.

### 2.2. Search Strategy, Information Sources and Data Management

The search strategy was developed by the systematic review team (KB, JAB, JB, JD) and an experienced research librarian. Although the aim of this review was to investigate the benefit of including biofeedback in BrPT broadly, search terms relating to BrPD were included in the search strategy as BrPT is often the first‐line treatment approach in this population and the research team felt it was appropriate to specifically include. Full search strategies are available in Supporting Information 2. On January 31, 2024, KB performed an electronic search of databases, registries and archives from date of inception, with an updated search on May 09, 2024, and a final search on May 22, 2025. A summary of information sources is available in Supporting Information 3. All citations identified from the literature search were uploaded to a web‐based tool—Rayyan (https://www.rayyan.ai), allowing all citations to be managed on one platform with reviewer blinding for screening.

### 2.3. Selection Process

KB performed the initial screen of the information sources. Possible duplicate studies were identified by Rayyan and reviewed and deleted by KB. KB and JAB independently screened the identified abstracts and titles and subsequent full‐text articles. Any disagreement at both title/abstract screening and full‐text stage review was resolved by discussion between the two reviewers. Any conflict between the two independent reviewers was resolved by discussion and assessment of the study in question by a third reviewer (JD) and the decision to include made by consensus. Where possible, contact with the corresponding author(s) was made if clarification around study content was required.

### 2.4. Data Collection Process and Data Items

Data extraction was performed by KB using a study‐specific data extraction form and reviewed and agreed by the systematic review team. Data extracted consisted of first author; year of journal publication; journal title; study title; language of publication and country of origin; study setting; subject population/diagnosis; sample size; subject age and sex; type of biofeedback used; study design; study aims; study protocol; study outcomes; and study results and study conclusions. Study outcomes were collected as reported in the synthesised studies and separated into five main subgroups for discussion: respiratory outcomes; breathing patterns; hyperventilation syndrome (HVS) symptoms; QOL measures and functional performance indicators.

### 2.5. Risk of Bias in Individual Studies

Risk of bias in the included studies was assessed using the Cochrane risk of bias tool for randomised studies (RoB 2) [55] and the Cochrane risk of bias tool in nonrandomised studies of interventions (ROBINS I) [56]. Algorithms within the short version crib sheet were used to guide judgement pathways for RoB 2 assessments [57], and a detailed guidance document was used to guide judgement pathways for ROBINS I assessments [58]. Risk of bias assessments were applied independently to all included studies and were performed by KB and JAB. Any inconsistencies in assessment outcome were resolved by discussion to reach agreement between the two reviewers. A third reviewer was available if consensus could not be reached (JB). The main outcomes for all the included studies were assessed, and a judgement of risk of bias for each domain was made.

### 2.6. Data Synthesis

Clinical and methodological heterogeneity of the included studies was assessed by examining study designs, populations, interventions and outcomes. Customised tables were created to support analysis, and the research team drew on their knowledge and experience to determine heterogeneity by consensus. Due to the differences in synthesised study characteristics and low pooled sample numbers and to achieve the study’s exploratory objectives, a narrative synthesis was completed. Data synthesis was additionally guided by the synthesis without meta‐analysis (SWiM) in systematic reviews reporting guideline [59]. Key findings and overall evidence for each outcome have been analysed and presented using structured summarised text and summary of findings tables, with data presented as mean values ± standard deviation. If statistical significance was reported, standardise effect size using bias‐corrected Hedges *g* was computed. Confidence intervals for effect size were calculated based on the mean and standard deviation differences.

### 2.7. Certainty Assessment

The quality and certainty of evidence for each study were independently assessed by two reviewers (KB and JD) using the Grading of Recommendations Assessment, Development and Evaluation (GRADE) criteria [60].

## 3. Results

Inter‐rater reliability for KB and JAB was investigated prior to the start of abstract and title screening to quantify agreement. A kappa co‐efficient value of 0.78 was calculated, suggesting substantial inter‐rater agreement [61]. Please see Supporting Information 4 for further details. A total of 1196 studies were identified from information sources, of which 794 articles were screened at the title and abstract stage. One study could not be found, and two studies were excluded due to an inadequate language translation. Four studies were referred to the third reviewer, of which three were included in the final synthesis. In total, 103 full‐text articles were reviewed and 8 studies [50, 62–68] included in this review. The screening process is outlined in Figure 1, and the included studies are reported and summarised in Tables 1 and 2.

**FIGURE 1:**
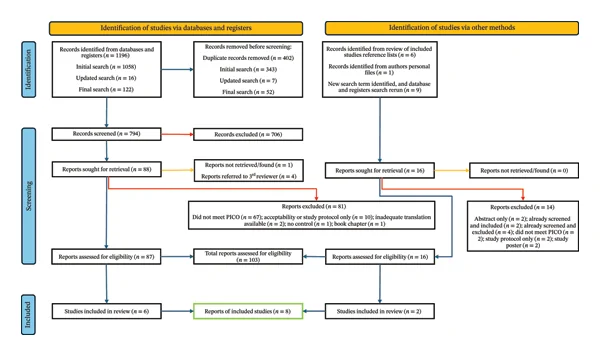
Flow diagram of the screening process.

**TABLE 1:** Summary of data items.

First author	Year of publication	Journal title	Study title	Language of publication and country of origin	Study setting and response	Response to biofeedback (acute or training)	Participant population/diagnosis	Sample size	Mean age (SD) in years: sex
Ishii	2021	Journal of Bodywork and Movement Therapies	Effects of visual feedback of thoracoabdominal motion on oxygen consumption during hyperventilation‐Pilot study	English: Japan	Laboratory	Acute	Healthy	8	22.1 (3.3) M = 8

Smyth	2021	Sensors	Novel real‐time OEP phase angle feedback system for dysfunctional breathing pattern training‐An acute intervention study	English: United Kingdom	Laboratory	Acute	Suspected dysfunctional breathing patterns	18 Control *n* = 9 Experimental *n* = 9	Control: 28.6 (10.7) Experimental: 25.0 (8.2) Sex: unknown

van Gestel	2012	Respiration	The effects of controlled breathing during pulmonary rehabilitation in patients with COPD	English: Germany	Outpatient clinic and home based	Training	Stable COPD	40 Control *n* = 20 Experimental *n* = 20	Control: 66 (7.4) M = 8 and F = 12 Experimental: 66.3 (5.6) M = 9 and F = 11

Collins	2008	American Journal of Respiratory and Critical Care Medicine	Can ventilation‐feedback training augment exercise tolerance in patients with Chronic Obstructive Pulmonary Disease?	English: United States of America	Laboratory	Training	Moderate to severe COPD	64 randomised to three groups 49 completed VFE *n* = 17 VF *n* = 16 E *n* = 16	VFE: 68 (9) VF: 69 (7) E: 65 (7) Sex: Unknown

Collins	2003	Journal of Rehabilitation Research and Development	Effect of ventilation‐feedback training on endurance and perceived breathlessness during constant work‐rate leg‐cycle exercise in patients with COPD	English: United States of America	Laboratory	Training	Moderate to severe COPD	47 enrolled 39 completed VFE *n* = 13 VF *n* = 14 E *n* = 13	VFE: 68.9 (9.3) VF: 67.8 (7.6) E: 65.9 (7.6) Sex: Unknown

Estève	1996	Biofeedback and Self‐Regulation	The effects of breathing pattern training on ventilatory function in patients with COPD	English: France	In‐patient: pulmonary rehabilitation centre	Training	Stable COPD	19	BrPT: 66 (13) Control: 77 (8) Sex: unknown

Sackner	1984	American Journal of Respiratory and Critical Care Medicine	Effects of abdominal and thoracic breathing on breathing pattern components in normal subjects and in patients with COPD	English: United States of America	Laboratory	Acute	Healthy and COPD	19 Healthy *n* = 10 COPD *n* = 9	Healthy: 28 (4) M = 3 and F = 7 COPD: 59 (9) M = 3 and F = 6

van Doorn	1982	Bulletin européen de physiopathologie respiratoire	Control of the end‐tidal PCO_2_ in the hyperventilation syndrome: effects of biofeedback and breathing instructions compared.	English: Neverlands	Laboratory and home based	Training	Chronic hyperventilation syndrome	20	39 (10.6) M = 11 F = 9

*Note:* E = exercise alone; F = female; M = male; *n* = number; PCO_2_ = partial pressure of carbon dioxide; VF = ventilation feedback alone; VFE = ventilation feedback plus exercise.

Abbreviations: BrPT = breathing pattern training; COPD = chronic obstructive pulmonary disease; OEP = optoelectronic plethysmography.

**TABLE 2:** Summary of included studies.

Study	Type of biofeedback used	Study design	Study aims	Study protocol^∗^	Main measurements	Study results	Study conclusions
Ishii et al, 2021	Visual feedback displayed via Konno–Mead diagrams Ratio of thoracoabdominal contribution to tidal volume for each breath displayed	Pilot: Nonrandomised	To examine the influence of visual feedback of thoracoabdominal movement during diaphragmatic breathing on oxygen consumption in healthy men	Subjects practiced diaphragmatic breathing alone and with visual feedback for about 3 minutes Oxygen consumption measured for 10 min during quiet breathing; during diaphragmatic breathing for 3 min; during diaphragmatic breathing with visual feedback for 3 min	• RR • RatioTH‐ABD • VT • VE • Oxygen consumption (VO_2_/BW)	No significant difference in RatioTH‐ABD and ventilatory parameters between diaphragmatic breathing and diaphragmatic breathing with visual feedback VO_2_/BW decreased with visual feedback by an average of 0.6 mL/kg (*p* < 0.05)	Visual feedback of thoracoabdominal movement during diaphragmatic breathing may reduce respiratory muscle oxygen consumption Greater variability of thoracoabdominal movement without visual feedback potentially increasing respiratory muscle work and VO_2_/BW

Smyth et al, 2021	Visual feedback of volume via real‐time total volume trace plot and phase angle feedback via real‐time Konno–Mead loops	RCT	To investigate the use of a newly developed real‐time OEP phase angle feedback system as an acute breathing retraining intervention	Incremental cycle exercise test and prestudy measures taken Acute breathing retraining instruction for all participants Random allocation to the control group (volume‐based OEP feedback) or experimental group (volume‐based OEP feedback and phase angle OEP feedback) Repeated incremental cycle test and poststudy measures taken	• RR • RcAbPhase • RCpAbPhase • AbsPhase	Improved synchrony in the breathing pattern in the experimental group Experimental group showed an increase in RcAbPhase, RCpAbPhase and AbPhase phase angle postintervention with high‐intensity exercise and recovery During high‐intensity exercise and recovery RR decreased significantly postintervention in both groups	Dysfunctional breathing patterns can be acutely improved with real‐time OEP phase angle feedback

van Gestel et al, 2012	Visual display of the desired respiratory pattern and acoustic tones	RCT	To investigate whether controlled breathing improves outcome of rehabilitation in COPD patients	Outcomes tested prior to and following completion of cardiopulmonary exercise training Random allocation to the control group (conventional pulmonary rehabilitation) or experimental group (conventional pulmonary rehabilitation plus respiratory feedback training)	• Pulmonary function, including: FEV_1_; FVC; FEV_1_ (%pred); FVC (% pred) • Exercise capacity (6MWD) • Perceived breathlessness (CRDQ) • Perceived fatigue (CRDQ) • Emotional function (CRDQ) • Mastery (CRDQ)	No significant between‐group differences reported in pulmonary function, exercise capacity or health‐related quality of life	Controlled breathing using respiratory feedback training has no effect on pulmonary function, cardiopulmonary exercise capacity or health‐related quality of life

Collins et al, 2008	Visual feedback of inhalation and exhalation based on minimum expiratory flow rate, Te:Ti ratio and RR. Displayed as a bar that moved left during inhalation and right during exhalation	RCT	To determine whether patients with COPD would achieve longer exercise duration if randomised to a combination of exercise training plus ventilation feedback than either form of training on its own Secondary aims were to establish whether ventilation‐feedback training would alter respiratory pattern, reduce dynamic hyperinflation and reduce perceived dyspnoea during treadmill exercise testing	Baseline exercise testing Random allocation VFE or E or VF 18 training sessions Repeat exercise testing 18 training sessions Repeat exercise testing	• Oxygen consumption (VO_2_ mL/kg/min) • VE • VT • RR • Ti • Te • IC • Perceived breathlessness (BORG) • Perceived fatigue (CRDQ) • Emotional function (CRDQ) • Mastery (CRDQ) • Exercise duration (treadmill)	Significant changes in oxygen consumption, VE, RR, Ti, Te, IC, perceived breathlessness and mastery with VFE Significant changes in oxygen consumption, RR and perceived breathlessness with E Significant changes to oxygen consumption, VE, RR, Ti, Te and perceived breathlessness and emotional function with VF Exercise duration improved in all groups. Greater in VFE.	The combination of VFE training decreases exercise‐induced dynamic hyperinflation and increase exercise duration more than VF alone Patients randomised to ventilation feedback with or without exercise were able to modify their breathing pattern All three groups reported a clinically significant reduction in dyspnoea

Collins et al, 2003	Visual feedback of inhalation and exhalation plus audible tones A horizontal bar for inspiration and expiration extended with the length of time spent in each respiratory phase Audible tones signified correct respiratory cycles achieved	RCT	To evaluate the efficacy of ventilation‐feedback training combined with leg‐cycle exercise to improve exertional endurance and decrease perceived dyspnoea in patients with COPD	Baseline exercise testing Random allocation to a 6‐week experimental intervention. Either VFE or E or VF only Repeat exercise testing	• Oxygen consumption (VO_2_ mL/min) • VE • VT • RR • Ti • Ti/Ttot • Te • IC • HR • Perceived breathlessness (BORG) • Perceived exertion (BORG) • Exercise duration (cycle)	Significant changes in VE, RR, Te, IC and perceived breathlessness with VFE Significant changes in RR and perceived breathlessness and exertion with E No significant changes in ventilatory parameters with VF only Exercise training effects seen across all 3 groups. The VFE and E groups significantly increased exercise duration from baseline VFE improved exercise tolerance sig more than those assigned to the VF only	Ventilation feedback with exercise and exercise only were equally effective in improving leg‐cycle exercise tolerance

Estève et al, 1996	Visual feedback of volume versus time Target and current breath displayed	Pilot study: RCT	To assess the effects of breathing pattern training on FEV_1_ and FVC in patients with COPD	Random allocation BrPT or control group RIP via sleeveless jacket applied. 10‐min familiarisation period of quiet breathing Breathing then recorded for 30 s. Subjects also concurrently enrolled on PR programme	• FEV_1_ • FVC • FEV_1_ (%pred) • FVC (% pred)	Significant increase in FEV_1_ and FVC in BrPT group	COPD patients trained to adjust their breath pattern to that of a longer and deeper pattern improve their FEV_1_ and FVC

Sackner et al, 1984	Visual feedback Ribcage versus abdominal loops	Comparative observational study	To assess the effect of voluntarily controlled breathing manoeuvres on breathing pattern components	Normal subjects: Supine: normal breathing for 10 min, abdominal breathing for 10 min, abdominally with visual feedback for 10 min, thoracically for 10 min and thoracically with visual feedback for 10 mins Patients with COPD: Supine: normal breathing for 10 min, abdominal breathing for 10 min, abdominal breathing with visual feedback for 10 min	• RR • VT • VE • VT/Ti • Ti • Ti/Ttot • %RC/VT • VTE/Te	Normal subjects: Expected changes were seen with the breathing manoeuvres Patients with COPD: heightened baseline respiratory centre drive compared to normal subjects evidenced by increased VE and VT/Ti	No significant improvement to breathing pattern with feedback in normal subjects Improved abdominal contribution to VT with feedback in subjects with COPD

van Doorn et al, 1982	Visual feedback‐visual peaks on a recorder and auditory feedback	Presumed convenience sample from outpatients from internal medicine and psychiatry departments RCT	To evaluate biofeedback training to raise end‐tidal PCO_2_ compared to breathing exercises/instruction	Baseline assessment Five‐week no treatment period Patients matched for end‐tidal PCO_2_ and random allocation to biofeedback training or breathing exercises Training regime implemented End‐tidal PCO_2_ measured at beginning of each treatment session, at pre‐ and post‐treatment evaluation and at 3‐month follow‐up	• End‐tidal PCO_2_ • Frequency of HVS symptoms	Both groups raised end‐tidal PCO_2_ levels. No significant differences in the treatment effect between the two groups Post‐treatment significant reduction in symptom complaint in the biofeedback group but not maintained at follow‐up Nonexperimental variable also analysed: pretreatment end‐tidal PCO_2_ levels: the higher the initial end‐tidal PcO_2_ level, the more the complaints decreased. The greater the rise in end‐tidal PCO_2_, the more the complaints decreased. Postintervention, the more the end‐tidal PCO_2_ levels rise, the more the complaints returned	HVS symptom complaint was reduced in the biofeedback group in the short term

*Note:* %RC/VT = percent ribcage to tidal volume, 6MWD = 6‐min walking distance, AbPhase = OEP phase angle feedback between the abdomen and shoulder compartments, BORG = rating of perceived breathlessness and exertion scale, *E* = exercise only, FEV_1_ = forced expiratory volume in 1 s, PCO_2_ = partial pressure of carbon dioxide, RatioTH‐ABD = ratio of thoracoabdominal contribution to tidal volume, RcAbPhase = OEP phase angle feedback between ribcage and abdomen, RCpAbPhase = OEP phase angle feedback between the pulmonary ribcage and the combined abdominal ribcage and abdomen, Te = expiratory time, Ti = inspiratory time, Ti/Ttot = fractional inspiratory time, VE = minute ventilation, VF = ventilation feedback only, VFE = ventilation feedback plus exercise, VO_2_/BW = oxygen consumption per body weight, VO_2_ mL/kg/min = oxygen consumption per mL/kilogram/minute, VO_2_ mL/min = oxygen consumption per mL/minute, VT = tidal volume, VT/Ti = mean inspiratory flow, VTE/Te = mean expiratory flow.

Abbreviations: BrPT = breathing pattern training, COPD = chronic obstructive pulmonary disease, CRDQ = chronic respiratory disease questionnaire, FVC = forced vital capacity, HR = heart rate, HVS = hyperventilation syndrome, IC = inspiratory capacity, OEP = optoelectronic plethysmography, RCT = randomised controlled trial, RIP = respiratory inductive plethysmography, RR = respiratory rate, PR = pulmonary rehabilitation.

^∗^Protocol summarised further in Supporting Information 5.

### 3.1. Author and Journal Characteristics

Research teams presented in this systematic review were from Japan [65], the United Kingdom [50], Germany [68], the United States of America [62, 63, 66], France [64] and the Netherlands [67]. All studies were presented in English. Publication date ranged from 1982 to 2021.

### 3.2. Study Characteristics

Six studies were randomised controlled trials (RCTs) [50, 62–64, 67, 68], and two studies were nonrandomised controlled trials (NRCTs) [65, 66]. Of the two NRCTs, one was a pilot study [65] and one used a convenience sample without randomisation [66]. Three studies were an acute intervention in a laboratory‐based setting [50, 65, 66] and studied the immediate effect of biofeedback. Three studies used a combination of laboratory‐based and home‐based settings [62, 63, 67], over a period of up to 7 weeks, with a 3‐month follow‐up [67]. One was a combination of an outpatient clinic setting and home‐based settings [68], with intervention over 4 weeks. One was based in an in‐patient pulmonary rehabilitation centre [64], with intervention over 4 weeks. Five studies therefore investigated the training effects of biofeedback. The studies included in this review describe the short‐term effects of biofeedback on outcomes, and the long‐term effects of biofeedback in BrPT are not evidenced.

### 3.3. Participant Characteristics

In total, 212 participants were included across eight studies. Participants were reported to be healthy subjects (*n* = 18; 2 studies); mean age 25 ± 3.65 years, subjects with suspected BrPD (*n* = 18; 1 study); mean age 26.8 ± 9.45 years, subjects with stable COPD (*n* = 156; 5 studies); mean age 67.17 ± 8.22 years or subjects presenting with chronic HVS (*n* = 20; 1 study); and mean age 39 ± 10.6 years. One study included both healthy and stable COPD participants, hence why study number in brackets equals 9 rather than 8. Whilst paediatric and adult populations were included in the initial screening, the included studies were all in adult populations. None of the included studies had explicit exclusion criteria of ≤ 18 years, although the included pathological groups would likely exclude paediatric cohorts.

### 3.4. Biofeedback Characteristics

All eight studies used visual feedback (VF) with three studies using additional auditory feedback. Further protocol summaries are available in Supporting Information 5.

### 3.5. Risk of Bias and GRADE Assessments

Thirty individual risk of bias assessments were completed: twenty‐five RoB 2 and five ROBINS I. Sixteen of the RoB 2 assessments were judged to be of serious concern, and nine were judged to be of high risk. Three of the ROBINS I assessments did not have enough information to base a judgement on and two were judged to be of serious risk. Risk of bias is therefore a substantial concern in the synthesised studies. Please see Supporting Information 6 for individual outcome specific risk of bias assessments. GRADE ratings across all studies ranged from low to very low, indicating that the quality of the available evidence is limited. Summaries of the risk of bias and GRADE decision rationales are available in Supporting Information 7.

### 3.6. Outcomes

Studies were grouped by outcome and population to enable a more meaningful analysis. Twelve outcomes were identified based on the reported main outcomes of the synthesised studies and were agreed by the review authors. Table 3 and Figure 2 present the main evidence, including effect sizes.

**TABLE 3:** Summary of evidence.

	Breathing patterns	Respiratory symptoms	Respiratory outcomes					Quality of life measures				Functional performance indicators
Outcomes ⟶	TAM/CWS	HVS symptoms	Oxygen consumption	Ventilatory parameters	Inspiratory capacity	FEV_1_ and FVC	End‐tidal PCO_2_	Perceived breathlessness	Perceived exertion/fatigue	Emotion	Mastery	Exercise capacity/duration

Population ↓												
Healthy	⊕ 1 NRCT 1 OS *n* = 8 [65] Δ [66] Δ	No evidence	⊕ 1 NRCT [65] *n* = 8 ¥Ⓜ	⊕ 1 NRCT 1 OS *n* = 18 [65] Δ [66] Δ	No evidence	No evidence	No evidence	No evidence	No evidence	No evidence	No evidence	No evidence

Suspected BrPD	⊕ 1 RCT [50] *n* = 18 ^∗^Ⓛ	No evidence	No evidence	⊕ 1 RCT [50] *n* = 18 ^∗^RRRⓈ	No evidence	No evidence	No evidence	No evidence	No evidence	No evidence	No evidence	No evidence

COPD	⊕ 1 RCT [66] *n* = 9 ¥Ⓜ	No evidence	⊕ 2 RCTs *n* = 88 [62] ⌂Ⓢ [63] ¤Δ	⊕ 2 RCTs 1 OS *n* = 97 [62] ⌂×VEⓂ ⌂RRⓂ ×RRⓂ ⌂TiⓁ: ×TiⓂ ⌂TeⓁ: ×TeⓁ [63] ⌂VEⓈ: ⌂RRⓂ ⌂TeⓈ [66] ¤ Δ	⊕⊕ 2 RCTs *n* = 88 [62] ⌂Ⓜ [63] ⌂Ⓢ	⊕ 2 RCTs *n* = 59 [64] ^∗^Ⓛ [68] ¤	No evidence	⊕ 3 RCTs *n* = 128 [62] ¤ [63] ⌂Ⓜ [68] ¤	⊕ 3 RCTs *n* = 128 [62,63,68] ¤Δ	⊕ 2 RCTs *n* = 89 [62] † [68] ¤	⊕ 2 RCTs *n* = 89 [62] ҂ [68] ¤	⊕ 3 RCTs *n* = 128 [62] ⌂Ⓛ [63] ⌂ [68] ¤

HVS	No evidence	⊕ 1 RCT [67] *n* = 20 ^∗^Ⓢ	No evidence	No evidence	No evidence	No evidence	⊕ 1 RCT [67] *n* = 20 ¤	No evidence	No evidence	No evidence	No evidence	No evidence

*Note:* FEV_1_ = forced expiratory volume in 1 s, HVS = hyperventilation syndrome, *n* = subject number, PCO_2_ = partial pressure of carbon dioxide, RRR = respiratory rate at rest, Te = expiratory time, Ti = inspiratory time, TAM/CWS = thoracoabdominal movement/chest wall synchrony, VE = minute ventilation, Ⓛ = large effect size, Ⓜ = medium effect size, Ⓢ = small effect size, ^∗^ = between‐group difference, ¤ = no between‐group difference, ¥ = within‐group difference, ⌂ = within‐group difference (plus exercise), × = within‐group difference (feedback alone), Δ = no within‐group difference, † =  within‐group change to MCID (feedback alone), ҂ = within‐group change to MCID (plus exercise). ⊕ = Very low quality/certainty, ⊕⊕ = low quality/certainty, ⊕⊕⊕ = moderate quality/certainty, ⊕⊕⊕⊕ = high quality/certainty (GRADE rating). [50] Smyth et al. 2021, [62] Collins et al. 2008, [63] Collins et al. 2003, [64] Estève et al. 1996, [65] Ishii et al. 2021, [66] Sackner et al. 1984, [67] Van Doorn et al. 1982, [68] van Gestel et al. 2012.

Abbreviations: FVC = forced vital capacity, NRCT = nonrandomised controlled trial, OS = observational study, RCT = randomised controlled trial, RR = respiratory rate.

**FIGURE 2:**
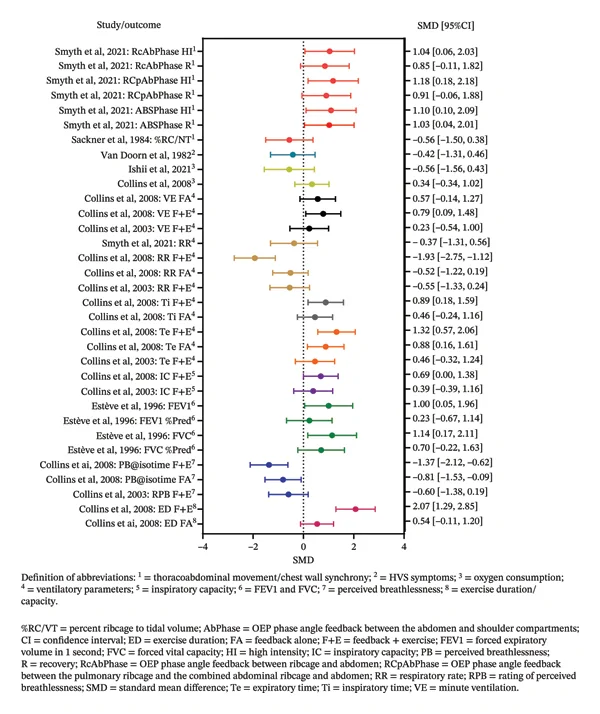
Standardised mean differences for outcomes demonstrating statistically significant effects of biofeedback.

### 3.7. Thoracoabdominal Movement (TAM)/Chest Wall Synchrony (CWS)

Three studies [50, 65, 66] (*n* = 45) investigated the effect of VF on TAM and CWS, using varied measures including OEP compartmental phase angles, thoracic excursion movement to total thoracic–abdominal movement and percentage of ribcage movement to tidal volume. Smyth et al. [50] reported statistically significant increases in OEP phase angle feedback in the experimental group pre‐to‐postintervention with high‐intensity exercise and with recovery, respectively. Improvements were observed between the ribcage and abdomen (RcAbPhase) (*p* < 0.01; *p* < 0.01), the pulmonary ribcage and the combined abdominal ribcage and abdomen (RCpAbPhase) (*p* < 0.01; *p* < 0.01) and between the abdomen and shoulder compartments (AbSPhase) (*p* < 0.01; *p* < 0.01).

Sackner et al. [66] reported an improvement in percent ribcage contribution to tidal volume in both groups across all breathing manoeuvres. Although no significant effect was seen in normal subjects, COPD patients demonstrated a greater abdominal compartment contribution (77.1% compared to 69% without VF (*p* < 0.05). Ishii et al. [65] reported no significant difference in the ratio of thoracoabdominal contribution to tidal volume (RatioTH‐ABD) with VF, although nonsignificant improvements were noted.

### 3.8. HVS Symptoms

One study (*n* = 20) investigated the effect of biofeedback on HVS symptoms [67]. HVS symptoms in this study were based on the frequency of sixteen complaints measured on a 5‐point scale. A statistically significant improvement in symptoms postintervention was seen in the experimental group, which were not maintained at a 3‐month follow‐up (F value [1.19 = 5.56] *p* < 0.05).

### 3.9. Oxygen Consumption

Three studies (*n* = 96) investigated the effect of biofeedback on oxygen consumption, with conflicting results. Ishii et al. [65] reported an improvement in oxygen consumption with biofeedback; average oxygen uptake per body weight (VO_2_/BW [mL/min/kg]) during diaphragmatic breathing with VF was 0.6 mL/kg lower than during diaphragmatic breathing without VF (*p* < 0.03). Collins et al. [62] reported within‐group improvements in peak oxygen consumption (VO_2 peak_ [mL·kg^−1^·min^−1^]) in all groups, reaching significance in the feedback plus exercise group (*p* = 0.04) and the exercise‐only group (*p* = 0.05). Conversely, Collins et al. [63] found no significant impact of biofeedback on absolute oxygen consumption (VO_2_ [mL·min^−1^]).

### 3.10. Ventilatory Parameters

Five studies (*n* = 133) assessed the impact of biofeedback on ventilatory parameters. Four studies (*n* = 115) evaluated tidal volume (VT) and reported no significant changes, whilst conflicting results were reported in minute ventilation (VE). Collins et al. [62] reported within‐group improvements in VE with reductions of 15% (*p* < 0.01) for feedback alone and 23% (*p* < 0.05) for feedback plus exercise. Similarly, Collins et al. [63] reported no between‐group differences in VE but noticed a within‐group reduction of 9% (*p* < 0.05) in the feedback plus exercise group. In contrast, Ishii et al. [65] and Sackner et al. [66] reported no significant changes to VE with VF.

Respiratory rate (RR) was investigated in five studies (*n* = 133). Smyth et al. [50] reported a reduction in RR at rest in the experimental group (*p* < 0.05) pre‐to‐postintervention. Collins et al. [62, 63] reported no significant between‐group differences in RR but reported within‐group reductions. In the feedback plus exercise groups, reductions of 24% (*p* < 0.01) and 7% (*p* < 0.05) were observed, respectively. Feedback alone reduced RR by 13% (*p* < 0.01) [62]. Conversely, Ishii et al. [65] and Sackner et al. [66] reported no statistically significant change to RR with VF.

Inspiratory time (Ti) was investigated in three studies (*n* = 107). Collins et al. [62] reported a within‐group increase in Ti of 23% with feedback plus exercise (*p* < 0.01) and 13% in the feedback alone group (*p* < 0.05). In contrast, Collins et al. [63] and Sackner et al. [66] found no statistically significant change to Ti between or within groups. Expiratory time (Te) was investigated in two studies by the same research team (*n* = 88). Te increased by 36% (*p* < 0.01) in the feedback plus exercise group and by 34% (*p* = 0.01) with feedback alone [62] and by 10% (*p* = 0.04) in the feedback plus exercise group [63]. No significant changes in the ratio of inspiratory time to total breathing cycle time (Ti/Ttot) with VF were reported in two studies (*n* = 58) [63, 64]. Sackner et al. [66] found no significant effect of VF on inspiratory flow (VT/Ti) or expiratory flow (VTE/Te) (*n* = 19), although nonsignificant within‐group improvements were seen in normal subjects.

### 3.11. Inspiratory Capacity (IC)

Two studies (*n* = 88) investigated the effect of biofeedback on IC as a determinant for exercise‐induced dynamic hyperinflation. No significant between‐group difference in IC was reported [62, 63]. However, within‐group analyses revealed increases in IC. Collins et al. [62] found a mean increase of 0.54 (±0.43) L in the feedback plus exercise group (*p* < 0.01). Similarly, Collins et al. [63] reported a mean increase in IC of 0.3 (±0.8) L in the feedback plus exercise group (*p* < 0.05). Both studies reported no statistically significant change to IC in the feedback alone and exercise‐only groups.

### 3.12. Forced Expiratory Volume in 1 s (FEV_1_) and Forced Vital Capacity (FVC)

Two studies (*n* = 59) investigated the effect of VF on FEV_1_ and FVC, yielding conflicting results. Significant increases in FEV_1_ (*F* value [1.8 = 7.58] *p* < 0.03), percent predicted FEV_1_ (*F* value [1.8 = 6.09] *p* < 0.04), FVC (*F* value [1.8 = 7.65] *p* < 0.03) and percent predicated FVC (F value [1.8 = 7.70] *p* < 0.03) were reported in the intervention group [64]. In contrast, van Gestel et al. [68] reported no significant change in lung function in either the experimental or the control group.

### 3.13. End‐Tidal PCO_2_

One study (*n* = 20) investigated the effect of VF on end‐tidal partial pressure of carbon dioxide (PCO_2_) [67]. Both groups statistically significantly raised end‐tidal PCO_2_, but no significant difference in treatment effect was reported between the two groups.

### 3.14. Perceived Breathlessness

Three studies (*n* = 128) investigated the effect of biofeedback on perceived breathlessness in individuals with COPD. Van Gestel et al. [68] and Collins et al. [62] used the Chronic Respiratory Disease Questionnaire (CRDQ), to assess breathlessness during activities of daily living. Collins et al. [62, 63] used the BORG scale to measure acute levels of dyspnoea. Van Gestel et al. [68] reported within‐group improvements in QOL in both experimental and control groups (*p* < 0.01), but no between‐group difference in breathlessness (*p* = 0.14).

Collins et al. [62] measured perceived dyspnoea at isotime, defined as the time between the beginning of exercise testing and the last set of measurements before completion of testing. They reported within‐group improvements in perceived breathlessness in all intervention groups: a 77% reduction with feedback plus exercise (*p* < 0.01), 64% with exercise‐only (*p* < 0.01) and 42% with feedback alone group (*p* < 0.02). The proportion of participants who cited dyspnoea as the primary reason for stopping exercise decreased in all groups: from 53% to 18% in the feedback plus exercise group (*p* < 0.02), from 63% to 44% in the feedback alone group (*p* < 0.09) and from 50% to 25% in the exercise‐only group (*p* < 0.05). Improvements in dyspnoea during activities of daily living exceeded the CRDQ minimal clinically important difference (MCID) of 0.5 per item [69] in all groups.

Collins et al. [63] reported significant reductions in perceived breathlessness in the feedback plus exercise (*p* < 0.01) and the exercise‐only groups (*p* < 0.05), but not in the feedback alone group. When breathlessness was plotted against exercise time, there was a decrease in the slope for all interventions but only statistically significant in the exercise‐only group (*p* < 0.02). Area under the curve for reported breathlessness (AUC_RPB_) decreased in the feedback plus exercise group (*p* = 0.03) and the exercise‐only group (*p* < 0.02), with a nonsignificant decrease in the feedback alone group.

### 3.15. Perceived Exertion/Fatigue

Three studies (*n* = 128) investigated the effect of biofeedback on perceived exertion or fatigue in subjects with COPD. Van Gestel et al. [68] and Collins et al. [62] assessed fatigue using the CRDQ, and Collins et al. [63] utilised the BORG scale. No statistically significant change to fatigue scores with biofeedback was reported by both van Gestel et al. [68] (*p* = 0.14) and Collins et al. [62]. Similarly, Collins et al. [63] found nonsignificant decreases in perceived exertion in both feedback groups, although a statistically significant decrease was seen in the exercise‐only group (*p* < 0.05). When exertion was plotted against exercise time, there was a decrease in the slope for all interventions but only statistically significant in the exercise‐only group (*p* < 0.02). Area under the curve for perceived exertion (AUC_RPE_) significantly decreased in the feedback plus exercise group (*p* = 0.04) and in the exercise‐only group (*p* < 0.01), but not in the feedback alone group.

### 3.16. Emotional Function

Two studies (*n* = 89) investigated the effect of biofeedback on emotional function in subjects with COPD, yielding mixed results. Collins et al. [62] saw emotional function measured by the CRDQ increase (*p* = 0.05) in the feedback alone group, exceeding the MCID for the CRDQ [69]. Van Gestel et al. [68] reported no between‐group difference in emotional function (*p* = 0.29).

### 3.17. Mastery

Mastery was assessed in two studies (*n* = 89) in subjects with COPD using the CRDQ. Collins et al. [62] reported significant improvements in the feedback plus exercise group (*p* < 0.01), exceeding the MCID for the CRDQ [69]. Conversely, no between‐group difference (*p* = 0.30) was noted in the study by van Gestel et al. [68].

### 3.18. Exercise Duration/Capacity

Three studies (*n* = 128) assessed exercise duration or capacity using the 6‐min walking test [68], a symptom limited treadmill test [62] and a constant work‐rate leg‐cycle test [63]. Van Gestel et al. [68] reported no between‐group difference in 6‐min walking test (*p* = 0.16). In contrast, Collins et al. [62] reported a mean increase in the exercise duration in all intervention groups, among study completers (*n* = 49). Mean treadmill time increased by 29.5 min in the feedback plus exercise group (10.5 ± 5.1 to 40.0 ± 20.4), 7.1 min in the feedback alone group (9 ± 3.6 to 16.1 ± 19.3) and 21.4 min in the exercise‐only group (10.1 ± 5.7 to 31.5 ± 17.3). Improvements were greater in the feedback plus exercise group than in the feedback alone group (*p* < 0.01) or the exercise‐only group (*p* < 0.03) and greater in the exercise‐only group than in the feedback alone group (*p* < 0.01). Subgroup analysis suggested that subjects with FEV_1_ < 50% showed an end of training exercise duration of 36.7 ± 3.7 min (feedback plus exercise) 11.9 ± 4.6 min (feedback alone) and 24.1 ± 3.9 min (exercise only). The difference in the exercise duration at the end of training between feedback plus exercise and exercise only was 52% (*p* < 0.03). Subjects with a baseline FEV_1_ > 50% showed end of training exercise durations of 48.0 ± 7.6 min (feedback plus exercise), 20.4 ± 6.4 min (feedback alone) and 41.8 ± 7.2 min (exercise only), with no significant difference in the exercise duration between feedback plus exercise and exercise alone (*p* = 0.56). Disease severity may, therefore, be an interesting consideration in determining the type of intervention that would be most beneficial in this cohort. Preset exercise time was set at 60 min, but no patient reached this at baseline. After training, 41% of patients in the feedback plus exercise, 19% of patients in the exercise‐only group and no patient in the feedback‐alone group reached this. Collins et al. [63] reported within‐group improvements in cycle time. There was an increase of 11.5 min in exercise time in the feedback plus exercise group (103%), 0.4 min in the feedback‐alone group (4%) and 8 min in the exercise‐only group (66%). Cycling time at 6 weeks was significantly less in the feedback‐alone group compared to the feedback plus exercise group (*p* < 0.01). No significant difference was observed in exercise endurance between the feedback plus exercise and exercise‐only groups.

## 4. Discussion

This review provides a focussed synthesis of the evidence for the use of biofeedback in BrPT, highlighting the current evidence base. Whilst improvements in breathing pattern, symptom scores, respiratory measures and QOL have been highlighted, the evidence supporting this is of low‐quality and in limited populations. However, these findings justify further investigation into the use of biofeedback in BrPT, as its effectiveness remains uncertain.

### 4.1. Respiratory Outcomes

There appears to be an agreement within the included studies that biofeedback has a nonsignificant main effect on VT. This is important to consider as BrPT focusses in some part on depth of breathing and so if this is a goal within rehabilitation, biofeedback would likely offer little above conventional BrPT. Within‐group improvements were reported in VE by Collins et al. [62] in both feedback groups and by Collins et al. [63] in the feedback plus exercise group. Improvements in VE are likely due to the reported within‐group changes to RR, Ti, Te and IC (combined with exercise). Ishii et al. [65] report improvements in oxygen consumption, but caution must be applied to this due to a small sample size of healthy participants (*n* = 8). In a pathological group, improvements in oxygen consumption seen by Collins et al. [62] demonstrated a small effect size. Improvements in reduction of RR have been outlined in this review. Studies investigating the use of biofeedback in HVS secondary to psychological disorder also describe a reduction in RR [70–72], which appears significant in this subset of BrPD. Reduction in the RR forms a major component of BrPT and is therefore crucial to rehabilitation. Additionally, this review has not demonstrated a significant influence of biofeedback on inspiratory or expiratory flow and further exploratory work is required.

The effect of biofeedback on FEV_1_ and FVC is not established, with a lack of high‐quality studies to support its use. Caution must be applied to the study by Estève et al. [64], as participants in this study were in‐patients in a pulmonary rehabilitation centre and had concurrent treatment with physiotherapy and bronchodilators. Although a control group was used, there is minimal documentation of these factors or a post hoc analysis. These concurrent treatment modalities may also have influenced lung function. Although the monitoring of end‐tidal PCO_2_ showed no between‐group difference in this review, the study investigators accepted that the two groups were not well matched for baseline end‐tidal PCO_2_ [67]. Improvements in PCO_2_ using capnography/capnometry are documented in HVS secondary to panic disorder [70–72] with sound rationale and therefore should be further explored.

### 4.2. Breathing Patterns

The measurement of breathing pattern is fundamental when investigating interventions addressing BrPT. Encouraging results were reported by Smyth et al. [50], who used a phase angle OEP feedback system with significant treatment effect on TAM and CWS. Other studies reported in this systematic review utilised respiratory inductance plethysmography to measure TAM and CWS, with less impact on outcomes. The implementation of novel approaches to both measure breathing pattern and for use with training could therefore be of importance when considering the incorporation of biofeedback in BrPT.

### 4.3. HVS Symptoms

HVS symptoms were improved immediately postintervention with biofeedback in the one study included in this review, although improvements were not maintained at 3‐month follow‐up. The use and benefits of biofeedback in HVS have been previously summarised [73], however, the studies outlined are largely in populations with anxiety‐ or panic‐related conditions rather than in the primary management of BrPD.

### 4.4. QOL Measures

Although within‐group improvements in QOL were noted in the included studies, no significant between‐group difference in perceived breathlessness was demonstrated with the addition of VF. However, van Gestel et al. [68] did report that participants had difficulty altering their breathing pattern during exercise, which might have affected outcomes. Benefits of biofeedback on perceived exertion and fatigue were also not established in this review. Interestingly, perceived breathlessness and mastery improved when biofeedback was combined with exercise [62, 63]. Of note, emotional function improved in the feedback‐alone group in the study by Collins et al. [63]. Further research investigating the effect of biofeedback on QOL measures is required.

### 4.5. Functional Performance Indicators

Increases in the exercise duration were seen in all groups in the studies by Collins et al. [62, 63], with additional benefits when combined with exercise. Exercise duration in these studies were examined during treadmill and cycle exercise. Further work investigating exercise duration would be valuable, particularly in sport specific activities and functional exercise.

Four distinct participant groups were investigated in the included studies: healthy participants, subjects with a suspected BrPD, stable and moderate to severe COPD and individuals with HVS. Sackner et al. [66] reported that the benefit of VF on RatioTH‐ABD was not significant in normal subjects but noticed significance in the COPD group. Whether biofeedback in BrPT favours pathological groups over healthy subjects is not clear, and further studies with clinical cohorts are required. Whilst it is acknowledged that pathological changes will occur in subjects with COPD due to the disease process, the included studies were evaluating the effect of biofeedback on outcomes in stable disease and not evaluating the effect of biofeedback on COPD management per se and are therefore relevant to include in this review. The combination of exercise and VF suggests greater improvements in VE, RR, Te, IC, exercise duration, perceived breathlessness and mastery. Therefore, combinations of intervention using biofeedback might be useful to explore for improved outcomes moving forward.

The use of respiratory biofeedback protocols features in published studies, but the focus of these has largely been on generalised outcomes or in the management of other pathologies and not in BrPT for BrPD specifically [36–42, 73]. Until now, the use of biofeedback in BrPT with a focus on BrPD has not been clearly mapped out in the literature. Given that BrPD is consistently described as a multidimensional disorder, with both physiological and psychological constructs [13, 17, 74, 75], the use of biofeedback could be particularly relevant in management strategies as the analysis of evidence suggests that it could be applied to rehabilitation within both physiological and psychological paradigms. This therefore strengthens the need for further studies investigating biofeedback in BrPT and the relevance of biofeedback to clinical practice.

### 4.6. Strengths and Limitations

The strengths of this review include the detailed methodology, the comprehensive search strategy and clearly defined inclusion and exclusion criteria. A thorough assessment of risk of bias and quality provides additional rigour. Our findings support previous work [73], which also identified significant heterogeneity and variable study quality in studies investigating interventions for BrPD. Bondarenko et al. [73] also point to a more significant within‐group change compared to a between‐group difference, supporting our findings. The authors of this review are aware that the low numbers of included studies, small sample sizes and general study heterogeneity are a limitation to study evaluation and make generalisation challenging. Two studies could not be fully assessed for inclusion due to an inadequate language translation and one study at the screening stage was unable to be found, which may have further informed the results. Initial screening and data extraction were performed by one reviewer (KB), which may have inadvertently increased the potential for screening or extraction error and reviewer bias. We were unable to perform a meta‐analysis due to inconsistency across the included studies. The variance and diversity in outcome measurement of the included studies and in how outcomes were defined or quantified also add to inconsistency. The psychophysiological aspects of BrPT were not examined in this review as they have been reported on by previous research groups, but this did limit the availability of appropriate studies for inclusion. For example, if the main reported outcomes relating to HVS were in the assessment of psychological disorders, the study was not included, as it was felt that this was a distinct subset population. Previous work has clearly detailed the use and benefits of biofeedback in stress and anxiety management [76, 77]. Additionally, the monitoring of carbon dioxide levels using biofeedback via capnography has been detailed in anxiety and panic disorders [78] and described in individuals with COPD [35] as part of a holistic intervention and during paced breathing in asthma [79]. Benefits in psychological measures, PCO_2_ measurements and RR have previously been shown [70–72]. The noninclusion of predominantly psychophysical uses of biofeedback in this review is not due to disregard of significance, as the research team acknowledges it to be clinically relevant and important, more so the use within this context is well documented.

BrPT is consistently reported in the management of BrPD, and establishing the effectiveness of biofeedback in populations with BrPD is of clinical relevance, whether presenting as a primary disorder or as a secondary sequelae. High‐quality and rigorous studies investigating the application and impact of biofeedback in BrPT are required. The focus of studies should be on determining both the acute effects and training responses, since the impact of both remains undetermined. Despite the current lack of evidence for its application in BrPT, the use of biofeedback could be particularly relevant in the management of BrPD if we consider the biomechanical, biochemical and psychophysiological classifications of BrPD summarised by Courtney [15]. From the studies included and highlighted, rationale for the use of biofeedback could be applied in all three of the above domains, making populations with BrPD ideal for future potential research studies.

## 5. Conclusion

Overall, there is a lack of reliable evidence of sufficient quality to suggest that the addition of biofeedback to BrPT provides greater benefits than conventional training or exercise alone. Improvements reported were more frequently observed within‐group than between‐group. Evidence for the use of biofeedback in BrPT is based on few studies and shows a high risk of bias, low level of certainty and quality, and substantial study heterogeneity. Critical challenges in interpreting the overall effectiveness of biofeedback in BrPT remain. Further studies investigating the application and impact of biofeedback within this area are required.

## Author Contributions

Kristopher Bahadur, Jake Bowd and John Dickinson made substantial contributions to the conception of the work. Kristopher Bahadur, Julie Backley, Jake Bowd and John Dickinson made significant contributions to the data analysis and interpretation. Kristopher Bahadur, Jake Bowd and John Dickinson made significant contributions to the design of the work and the interpretation of data. Julie Backley made significant contributions to the interpretation of data. Kristopher Bahadur drafted the original manuscript. Julie Backley, Jake Bowd and John Dickinson substantially contributed to the revision of the manuscript drafts.

## Funding

This systematic review was conducted as part of the corresponding authors’ PhD studentship, which was part funded by POWERbreathe International Ltd. Northfield Road, Southam, Warwickshire, CV47 0FG, and part funded by the University of Kent, Canterbury, UK. Kristopher Bahadur received a research stipend from the University of Kent and POWERbreathe International Ltd. John Dickinson received funding from POWERbreathe International Ltd. to support delivery of the PhD scholarship that Kristopher Bahadur is completing. POWERbreathe International Ltd. has not been involved in the conceptualisation, analysis, delivery, write‐up, or decision to publish this paper.

## Disclosure

All authors have approved the submitted version of the manuscript and agreed to be accountable for any part of the work.

## Conflicts of Interest

John Dickinson was a contributing author to one of the papers included in this systematic review [50] but was not involved in study screening, decision to include or risk of bias assessments. The other authors declare no conflicts of interest.

## Supporting Information

Additional supporting information can be found online in the Supporting Information section.

## Supporting information


**Supporting Information 1** PICO framework.

**Supporting Information 2** Full search strategy.

**Supporting Information 3** Summary of information sources.

**Supporting Information 4** Inter‐rater reliability.

**Supporting Information 5** Information for protocols.

**Supporting Information 6** Risk of bias per outcome.

**Supporting Information 7** RoB and GRADE decision rationale.

**Supporting Information 8** PRISMA_2020_checklist.

## Data Availability

The datasets generated or analysed during this study are included in this published article and its additional information files. Further clarification or details are available from the corresponding author on reasonable request.

## References

[1] Thomas M. , Mckinley R. K. , Freeman E. , Foy C. , and Price D. , The Prevalence of Dysfunctional Breathing in Adults in the Community With and Without Asthma, Primary Care Respiratory Journal. (2005) 14, no. 2, 78–82, 10.1016/j.pcrj.2004.10.007. PMC6743552 16701702

[2] Grammatopoulou E. , Skordilis E. , Georgoudis G. et al., Hyperventilation in Asthma: A Validation Study of the Nijmegen Questionnaire-NQ, Journal of Asthma. (2014) 51, no. 8, 839–846, 10.3109/02770903.2014.922190. 24823322

[3] Thomas M. , Mckinley R. K. , Freeman E. , and Foy C. , Prevalence of Dysfunctional Breathing in Patients Treated for Asthma in Primary Care: Cross Sectional Survey, BMJ. (2001) 322, no. 7294, 1098–1100, 10.1136/bmj.322.7294.1098. PMC31263 11337441

[4] Stanton A. E. , Vaughn P. , Carter R. , and Bucknall C. , An Observational Investigation of Dysfunctional Breathing and Breathing Control Therapy in a Problem Asthma Clinic, Journal of Asthma. (2008) 45, no. 9, 758–765, 10.1080/02770900802252093. 18972291

[5] Law N. , Ruane L. E. , Low K. , Hamza K. , and Bardin P. G. , Dysfunctional Breathing Is More Frequent in Chronic Obstructive Pulmonary Disease Than in Asthma and in Health, Respiratory Physiology & Neurobiology. (2018) 247, 20–23, 10.1016/j.resp.2017.08.011. 28870869

[6] Hull J. H. , Jackson A. R. , Ranson C. , Brown F. , Wootten M. , and Loosemore M. , The Benefits of a Systematic Assessment of Respiratory Health in Illness-Susceptible Athletes, European Respiratory Journal. (2021) 57, no. 6, 10.1183/13993003.03722-2020. 33334943

[7] Hull J. H. , Williams Z. , Jackson A. et al., Respiratory Problems in Rowers: Outcomes From a Systematic Assessment of an Elite Squad, British Journal of Sports Medicine. (2024) 0, no. 24, 1–8, 10.1136/bjsports-2024-108542. 39122371

[8] Gridina I. , Bidat E. , Chevallier B. , and Stheneur C. , Prevalence of Chronic Hyperventilation Syndrome in Children and Teenagers, Archives of Pediatrics. (2013) 20, no. 3, 265–268, 10.1016/j.arcped.2012.12.016. 23375424

[9] Han J. , Stegen K. , Simkens K. et al., Unsteadiness of Breathing in Patients With Hyperventilation Syndrome and Anxiety Disorders, European Respiratory Journal. (1997) 10, no. 1, 167–176, 10.1183/09031936.97.10010167. 9032511

[10] Jack S. , Rossiter H. B. , Warburton C. J. , and Whipp B. , Behavioural Influences and Physiological Indices of Ventilatory Control in Subjects With Idiopathic Hyperventilation, Behaviour Modification. (2003) 27, no. 5, 637–652, 10.1177/0145445503256318. 14531159

[11] Leyro T. M. , Versella M. V. , Yang M. J. , Brinkman H. R. , Hoyt D. L. , and Lehrer P. , Respiratory Therapy for the Treatment of Anxiety: Meta-Analytic Review and Regression, Clinical Psychology Review. (2021) 84, 10.1016/j.cpr.2021.101980. PMC8302658 33540222

[12] Frésard I. , Genecand L. , Altarelli M. et al., Dysfunctional Breathing Diagnosed by Cardiopulmonary Exercise Testing in ‘Long COVID’ Patients With Persistent Dyspnoea, BMJ Open Respiratory Research. (2022) 9, no. 1, 10.1136/bmjresp-2021-001126. PMC8968537 35354589

[13] Genecand L. , Altarelli M. , Binkova A. et al., Dysfunctional Breathing Symptoms, Functional Impact and Quality of Life in Patients With Long COVID-19: A Prospective Case Series, BMJ Open Respiratory Research. (2023) 10, no. 1, 10.1136/bmjresp-2023-001770. PMC10347459 37433720

[14] Clifton-Smith T. and Rowley J. , Breathing Pattern Disorders and Physiotherapy: Inspiration for Our Profession, Physical Therapy Reviews. (2011) 16, no. 1, 75–86, 10.1179/1743288X10Y.0000000025.

[15] Courtney R. , Breathing Training for Dysfunctional Breathing in Asthma: Taking a Multi-Dimensional Approach, ERJ Open Research. (2017) 3, no. 4, 00065–02017, 10.1183/23120541.00065-2017. PMC5721258 29234673

[16] Vidotto L. S. , Carvalho C. R. F. , Harvey A. , and Jones M. , Dysfunctional Breathing: What Do We Know?, Jornal Brasileiro de Pneumologia. (2019) 45, no. 1, 10.1590/1806-3713/e20170347. PMC6534396 30758427

[17] Barker N. and Everard M. , Getting to Grips With Dysfunctional Breathing, Paediatric Respiratory Reviews. (2015) 16, no. 1, 53–61, 10.1016/j.prrv.2014.10.001. 25499573

[18] Boulding R. , Stacey R. , Niven R. , and Fowler S. , Dysfunctional Breathing: A Review of the Literature and Proposal for Classification, European Respiratory Review. (2016) 25, no. 141, 287–294, 10.1183/16000617.0088-2015. PMC9487208 27581828

[19] Courtney R. , The Functions of Breathing and Its Dysfunctions and Their Relationship to Breathing Therapy, International Journal of Osteopathic Medicine. (2009) 12, no. 3, 78–85, 10.1016/j.ijosm.2009.04.002.

[20] Depiazzi J. and Everard M. , Dysfunctional Breathing and Reaching One’s Physiological Limit as Causes of Exercise Induced Dyspnoea, Breathe. (2016) 12, no. 2, 120–129, 10.1183/20734735.007216. PMC4933621 27408630

[21] Vlemincx E. , Dysfunctional Breathing: A Dimensional, Transdiagnostic Perspective, European Respiratory Journal. (2023) 61, no. 6, 10.1183/13993003.00629-2023. 37290807

[22] Hagman C. , Janson C. , and Emtner M. , A Comparison Between Patients With Dysfunctional Breathing and Patients With Asthma, The Clinical Respiratory Journal. (2008) 2, no. 2, 86–91, 10.1111/j.1752-699X.2007.00036.X. 20298312

[23] Chenivesse C. , Similowski T. , Bautin N. et al., Severely Impaired Health-Related Quality of Life in Chronic Hyperventilation Patients: Exploratory Data, Respiratory Medicine. (2014) 108, no. 3, 517–523, 10.1016/j.rmed.2013.10.024. 24269004

[24] Jones M. , Harvey A. , Marston L. , and O’Connell N. E. , Breathing Exercises for Dysfunctional Breathing/Hyperventilation Syndrome in Adults (Review), Cochrane Database of Systematic Reviews. (2013) 5, 10.1002/14651858.CD009041.pub2. PMC11379427 23728685

[25] Bott J. , Blumenthal S. , Buxton M. et al., Guidelines for the Physiotherapy Management of the Adult, Medical, Spontaneously Breathing Patient, Thorax. (2009) 64, no. suppl 1, i1–i51, 10.1136/thx.2008.110726. 19406863

[26] Hagman C. , Janson C. , and Emtner M. , Breathing Retraining-A Five-Year Follow-Up of Patients With Dysfunctional Breathing, Respiratory Medicine. (2011) 105, no. 8, 1153–1159, 10.1016/j.rmed.2011.03.006. 21454062

[27] Reilly C. , Floyd S. , Lee K. et al., Breathlessness and Dysfunctional Breathing in Patients With Postural Orthostatic Tachycardia Syndrome (POTS): The Impact of a Physiotherapy Intervention, Autonomic Neuroscience: Basic and Clinical. (2020) 223, 10.1016/j.autneu.2019.102601. 31743851

[28] Thomas M. , Mckinley R. , Freeman E. , Foy C. , Prodger P. , and Price D. , Breathing Retraining for Dysfunctional Breathing in Asthma: A Randomised Controlled Trial, Thorax. (2003) 58, 110–115, 10.1136/thorax.58.2.110. PMC1746567 12554890

[29] Denton E. , Bondarenko J. , O’Hehir R. , and Hew M. , Breathing Pattern Disorder in Difficult Asthma: Characteristics and Improvement in Asthma Control and Quality of Life After Breathing Re-Training, Allergy. (2019) 74, no. 1, 201–203, 10.1111/all.13611. 30243028

[30] Bondarenko J. , Bremner J. , Button B. , Hew M. , and Holland A. , Optimal Treatment for Dysfunctional Breathing in Adults: An International Delphi Study, Clinical and Experimental Allergy. (2026) 0, no. 5, 1–9, 10.1111/cea.70247. PMC13135875 41699370

[31] Manifield J. , Alexiou C. , Megaritis D. et al., Effects of Inspiratory Muscle Training on Thoracoabdominal Volume Regulation in Older Adults: A Randomised Controlled Trial, Respiratory Physiology & Neurobiology. (2024) 326, 10.1016/j.resp.2024.104278. 38735425

[32] Giggins O. , Persson U. , and Caulfield B. , Biofeedback in Rehabilitation, Journal of NeuroEngineering and Rehabilitation. (2013) 10, no. 1, 10.1186/1743-0003-10-60. PMC3687555 23777436

[33] Kondo K. , Noonan K. , Freeman M. , Ayers C. , Morasco B. , and Kansagara D. , Efficacy of Biofeedback for Medical Conditions: An Evidence Map 2019, Journal of General Internal Medicine. (2019) 34, no. 12, 2883–2893, 10.1007/s11606-019-05215-z. PMC6854143 31414354

[34] Schwartz M. S. , A New Improved Universally Accepted Official Definition of Biofeedback: Where Did It Come From? Why? Who Did It? Who Is It For? What’s Next?, Biofeedback. (2010) 38, no. 3, 88–90, 10.5298/1081-5937-38.3.88.

[35] Norweg A. , Hofferber B. , Oh C. et al., Capnography-Assisted Learned, Monitored (CALM) Breathing Therapy for Dysfunctional Breathing in COPD: A Bridge to Pulmonary Rehabilitation, Contemporary Clinical Trials. (2023) 134, 10.1016/j.cct.2023.107340. 37730198

[36] Costa V. J. E. , de Morais Nunes A. , de Albuquerque Cacique New York B. et al., Biofeedback Therapeutic Effects on Blood Pressure Levels in Hypertensive Individuals: A Systematic Review and Meta-Analysis, Complementary Therapies in Clinical Practice. (2021) 44, 10.1016/j.ctcp.2021.101420. 34062320

[37] Grossman E. , Grossman A. , Schein M. H. , Zimlichman R. , and Gavish B. , Breathing-Control Lowers Blood Pressure, Journal of Human Hypertension. (2001) 15, no. 4, 263–269, 10.1038/sj.jhh.1001147. 11319675

[38] Kapitza K. , Passie T. , Bernateck M. , and Karst M. , First Non-Contingent Respiratory Biofeedback Versus Contingent Biofeedback in Patients With Chronic Low Back Pain: A Randomized Controlled, Double-Blind Trial, Applied Psychophysiology and Biofeedback. (2010) 35, no. 3, 207–217, 10.1007/s10484-010-9130-1. 20237953

[39] Kaushik R. , Kaushik R. M. , Mahajan S. K. , and Rajesh V. , Biofeedback Assisted Diaphragmatic Breathing and Systematic Relaxation Versus Propranolol in Long Term Prophylaxis of Migraine, Complementary Therapies. (2005) 13, no. 3, 165–174, 10.1016/j.ctim.2005.04.004. 16150370

[40] Schein M. H. , Gavish B. , Herz M. et al., Treating Hypertension With a Device That Slows and Regulates Breathing: A Randomized, Double-Blind Controlled Study, Journal of Human Hypertension. (2001) 15, no. 4, 271–278, 10.1038/sj.jhh.1001148. 11319676

[41] Stanton R. , Ada L. , Dean C. M. , and Preston E. , Biofeedback Improves Performance in Lower Limb Activities More Than Usual Therapy in People Following Stroke: A Systematic Review, Journal of Physiotherapy. (2017) 63, no. 1, 11–16, 10.1016/j.jphys.2016.11.006. 27989731

[42] Wang R. , Zhang S. , Zhang J. et al., Electromyographic Biofeedback Therapy for Improving Limb Function After Stroke: A Systematic Review and Meta-Analysis, PLoS One. (2024) 19, no. 1, 10.1371/journal.pone.0289572. PMC10783731 38206927

[43] Chen M. A. , Spanton K. , Schaik P. , Spears I. , and Eaves D. , The Effects of Biofeedback on Performance and Technique of Boxing Jab, Perpetual and Motor Skills. (2021) 128, no. 4, 1607–1622, 10.1177/00315125211013251. PMC8267084 33940988

[44] Giraldo-Pedroza A. , Lee W. C. , Lam W. , Coman R. , and Alici G. , Effects of Wearable Devices With Biofeedback on Biomechanical Performance of Running-A Systematic Review, Sensors. (2020) 20, no. 22, 10.3390/s20226637. PMC7699362 33228137

[45] Pfeufer D. , Gililland J. , Böcker W. et al., Training With Biofeedback Devices Improves Clinical Outcome Compared to Usual Care in Patients With Unilateral TKA: A Systematic Review, Knee Surgery, Sports Traumatology, Arthroscopy. (2019) 27, no. 5, 1611–1620, 10.1007/s00167-018-5217-7. 30334150

[46] Shen W. , Yu Y. , Bocanegra J. F. , Wheeler P. C. , and Fong D. T. P. , Enhancing Running Injury Prevention Strategies With Real-Time Biofeedback: A Systematic Review and Meta-Analysis, Journal of Sports Sciences. (2024) 42, no. 110, 981–992, 10.1080/02640414.2024.2374637. 38967313

[47] Shokri A. and Nosratabadi M. , Comparison of Biofeedback and Combined Interventions on Athlete’s Performance, Applied Psychophysiology and Biofeedback. (2021) 46, no. 3, 227–234, 10.1007/s10484-020-09498-5. 33386459

[48] Tate J. J. and Milner C. E. , Real-Time Kinematic, Temporospatial, and Kinetic Biofeedback During Gait Retraining in Patients: A Systematic Review, Physical Therapy. (2010) 90, no. 8, 1123–1134, 10.2522/ptj.20080281. 20558567

[49] Wood C. M. and Kipp K. , Use of Audio Biofeedback to Reduce Tibial Impact Accelerations During Running, Journal of Biomechanics. (2014) 47, no. 7, 1739–1741, 10.1016/j.jbiomech.2014.03.008. 24679712

[50] Smyth C. , Winter S. , and Dickinson J. , Novel Real-Time OEP Phase Angle Feedback System for Dysfunctional Breathing Pattern Training-An Acute Intervention Study, Sensors. (2021) 21, no. 11, 10.3390/s21113714. PMC8199249 34073590

[51] Hmeidi H. , Motamedi-Fakhr S. , Chadwick E. et al., Tidal Breathing Parameters Measured Using Structured Light Plethysmography in Healthy Children and Those With Asthma Before and After Bronchodilator, Physics Reports. (2017) 5, no. 5, 10.14814/phy2.13168. PMC5350176 28275111

[52] Porras C. D. , Lunardi A. C. , Marques da Silva C. C. B. et al., Comparison Between the Phase Angle and Phase Shift Parameters to Assess Thoracoabdominal Asynchrony in COPD Patients, Journal of Applied Physiology. (2017) 122, 1106–1113, 10.1152/Japplphysiol.00508.2016. 28183817

[53] Pereira M. C. , Porras C. D. , Lunardi A. C. et al., Thoracoabdominal Asynchrony: Two Methods in Healthy, COPD, and Interstitial Lung Disease Patients, PLoS One. (2017) 12, no. 8, 10.1371/journal.pone.0182417. PMC5540557 28767680

[54] Page M. J. , Moher D. , Bossuyt P. M. et al., PRISMA 2020 Explanation and Elaboration: Updated Guidance and Exemplars for Reporting Systematic Reviews, BMJ. (2021) 372, 10.1136/bmj.n160. PMC8005925 33781993

[55] Sterne J. A. C. , Savović J. , Page M. J. et al., RoB 2: A Revised Tool for Assessing Risk of Bias in Randomised Trials, BMJ. (2019) 366, 10.1136/bmj.l4898. 31462531

[56] Sterne J. A. C. , Hernán M. A. , Reeves B. C. et al., ROBINS-I: A Tool for Assessing Risk of Bias in Non-Randomized Studies of Interventions, BMJ. (2016) 355, 10.1136/bmj.i4919. PMC5062054 27733354

[57] Higgins J. P. T. , Savović J. , Page M. J. , and Sterne J. A. C. , RoB 2 Development Group, 2019, 1–72, http://www.riskofbias.info/welcome/rob-2-0-tool/current-version-of-rob-2.

[58] Sterne J. A. C. , Higgins J. P. T. , Elbers R. G. , Reeves B. C. , and Development Group for ROBINS-I , Risk of Bias in Non-randomised Studies of Interventions (ROBINS-I): Detailed Guidance, 2016, http://www.riskofbias.info.

[59] Campbell M. , McKenzie J. E. , Sowden A. et al., Synthesis Without Meta-Analysis (SWiM) in Systematic Reviews: Reporting Guideline, BMJ. (2020) 368, 10.1136/bmj.l6890. PMC7190266 31948937

[60] Ryan R. and Hill S. , How to GRADE the Quality of the Evidence, 2016, Cochrane Consumers and Communication Group, https://cccrg.cochrane.org/author-resources.Version3.0.

[61] Landis J. R. and Koch G. G. , The Measurement of Observer Agreement for Categorical Data, Biometrics. (1977) 33, no. 1, 159–174, 10.2307/2529310. 843571

[62] Collins E. G. , Langbein E. W. , Fehr L. et al., Can Ventilation-Feedback Training Augment Exercise Tolerance in Patients With Chronic Obstructive Pulmonary Disease?, American Journal of Respiratory and Critical Care Medicine. (2008) 177, no. 8, 844–852, 10.1164/rccm.200703-477OC. 18202351

[63] Collins E. G. , Fehr L. , Bammert C. et al., Effect of Ventilation-Feedback Training on Endurance and Perceived Breathlessness During Constant Work-Rate Leg-Cycle Exercise in Patients With COPD, Journal of Rehabilitation Research and Development. (2003) 40, no. 5, 35–44, 10.1682/jrrd.2003.10.0035. 15074452

[64] Estève F. , Blanc-Gras N. , Gallego J. , and Benchetrit G. , The Effects of Breathing Pattern Training on Ventilatory Function in Patients With COPD, Biofeedback and Self-Regulation. (1996) 21, no. 4, 311–321, 10.1007/BF02214431. 9031710

[65] Ishii N. , Tomita K. , Takeshima R. et al., Effects of Visual Feedback of Thoracoabdominal Motion on Oxygen Consumption During Hyperventilation–Pilot Study, Journal of Bodywork and Movement Therapies. (2021) 28, 317–322, 10.1016/j.jbmt.2021.06.007. 34776158

[66] Sackner M. A. , Gonzalez H. F. , Jenouri G. , and Rodriguez M. , Effects of Abdominal and Thoracic Breathing on Breathing Pattern Components in Normal Subjects and in Patients With Chronic Obstructive Pulmonary Disease, American Journal of Respiratory and Critical Care Medicine. (1984) 130, no. 4, 584–587. 10.1164/arrd.1984.130.4.584 6486557

[67] Van Doorn P. , Folgering H. , and Colla P. , Control of End-Tidal PCO2 in the Hyperventilation Syndrome: Effects of Biofeedback and Breathing Instructions Compared, Bulletin Europeen de Physiopathologie Respiratoire. (1982) 18, no. 6, 829–836. 6821472

[68] van Gestel A. J. R. , Kohler M. , Steier J. , Teschler S. , Russi E. W. , and Teschler H. , The Effects of Controlled Breathing During Pulmonary Rehabilitation in Patients With COPD, Respiration. (2012) 83, no. 2, 115–124, 10.1159/000324449. 21474911

[69] Schünemann H. J. , Puhan M. , Goldstein R. , Jaeschke R. , and Guyatt G. , Measurement Properties and Interpretability of the Chronic Respiratory Disease Questionnaire, COPD: Journal of Chronic Obstructive Pulmonary Disease. (2005) 2, no. 1, 81–89, 10.1081/COPD-200050651. 17136967

[70] Meuret A. , Wilhelm F. , Ritz T. , and Roth W. , Feedback of End Tidal pCO2 as a Therapeutic Approach for Panic Disorder, Journal of Psychiatric Research. (2008) 42, no. 7, 560–568, 10.1016/j.jpsychires.2007.06.005. PMC2890048 17681544

[71] Meuret A. , Rosenfield D. , Seidel A. , Bhaskara L. , and Hofmann S. , Respiratory and Cognitive Mediators of Treatment Change in Panic Disorder: Evidence for Intervention Specificity, Journal of Consulting and Clinical Psychology. (2010) 78, no. 5, 691–704, 10.1037/a0019552. PMC3327286 20873904

[72] Tolin D. , McGrath P. , Hale L. , Weiner D. , and Gueorguieva R. , A Multisite Benchmarking Trial of Capnometry Guided Respiratory Intervention for Panic Disorder in Naturalistic Treatment Settings, Applied Psychophysiology and Biofeedback. (2017) 42, no. 1, 51–58, 10.1007/s10484-017-9354-4. PMC5344940 28194546

[73] Bondarenko J. , Burge A. T. , Bremner J. et al., Non-Pharmacological Interventions for Dysfunctional Breathing in Adults: A Systematic Review, Journal of Allergy and Clinical Immunology: In Practice. (2025) 13, no. 8, 2062–2074, 10.1016/j.jaip.2025.04.053. 40345332

[74] Jones M. , Troup F. , Nugus J. et al., Does Manual Therapy Provide Additional Benefit to Breathing Retraining in the Management of Dysfunctional Breathing? A Randomised Controlled Trial, Disability & Rehabilitation. (2015) 37, no. 9, 763–770, 10.3109/09638288.2014.941020. 25026508

[75] Steinmann J. , Lewis A. , Ellmers T. , Jones M. , MacBean V. , and Kal E. , Validating the Breathing Vigilance Questionnaire for Use in Dysfunctional Breathing, European Respiratory Journal. (2023) 61, no. 6, 10.1183/13993003.00031-2023. 37024133

[76] Banushi B. , Brendle M. , Ragnhildstveit A. et al., Breathwork Interventions for Adults With Clinically Diagnosed Anxiety Disorders: A Scoping Review, Brain Sciences. (2013) 13, no. 2, 10.3390/brainsci13020256. PMC9954474 36831799

[77] Morarend Q. , Spector M. , Dawson D. , Clark S. , and Holmes D. , The Use of a Respiratory Rate Biofeedback Device to Reduce Dental Anxiety: An Exploratory Investigation, Applied Psychophysiology and Biofeedback. (2011) 36, no. 2, 63–70, 10.1007/s10484-011-9148-z. 21365307

[78] Meckley A. , Balancing Unbalanced Breathing: The Clinical Use of Capnographic Biofeedback, Biofeedback. (2013) 41, no. 4, 183–187, 10.5298/1081-5937-41.4.02.

[79] Meuret A. E. , Rosenfield D. , Millard M. M. , and Ritz T. , Biofeedback Training to Increase PCO2 in Asthma With Elevated Anxiety: A One-Stop Treatment of Both Conditions, Psychosomatic Medicine. (2023) 85, no. 5, 440–448, 10.1097/PSY.0000000000001188. PMC10238676 36961348

