# Supplementary material for: Biofeedback in Breathing Pattern Training: A Systematic Review
Source: Can Respir J. 2026 Nov 15;2026:5567144. doi: 10.1155/carj/5567144 (PMC13770288; doi:10.1155/carj/5567144)
Supplement: Supplementary file 1 [file CARJ-2026-5567144-s001.docx]

| **Supplementary Material 2: Full search strategy** | **Date of Search** | **Search Number** | **Search Terms** Where possible, relevant MeSH terms and subject headings were incorporated into the search. | **Results** |
| --- | --- | --- | --- | --- |
| SPORTDiscus with full text  And  APA PsycInfo | 31/01/2024 | #1 | AB "real-time feedback" OR AB biofeedback OR AB "real-time biofeedback" OR AB "real-time proprioceptive feedback" OR AB "Real-time breath training" OR AB "real-time breath analysis" OR AB "real-time breath cycle analysis" OR AB "real-time breath regulation" OR AB "real-time breathing pattern training" OR AB feedback OR AB "breathing pattern training" OR AB "breathing training" **Expanders** - Apply equivalent subjects **Search modes** - Boolean/Phrase | 94,036 |
|  |  | #2 | AB "breathing pattern re-training" OR AB "breathing re-training" **Expanders** - Apply equivalent subjects **Search modes** - Boolean/Phrase | 4 |
|  |  | #3 | #1 or #2 | 94,038 |
|  |  | #4 | AB ( dysfunctional breathing pattern or dysfunctional breathing or breathing pattern disorder ) OR AB "breathing pattern dysfunction" OR AB "breathing dysfunction" OR AB "breathing patterns" OR AB hyperventilation OR AB ( hyperventilation syndrome or hyperventilation breathing ) OR AB "inducible laryngeal obstruction" OR AB "exercise induced laryngeal obstruction" OR AB "vocal cord dysfunction" OR AB "inspiratory stridor" OR AB "exercise induced paradoxical vocal fold motion" **Expanders** - Apply equivalent subjects **Search modes** - Boolean/Phrase | 2,129 |
|  |  | #5 | #3 and #4 | **101** |
|  | 09/05/2024 | #1 | "ventilation feedback training"" **Expanders** - Apply equivalent subjects **Search modes** - Boolean/Phrase | 0 |
|  |  | #2 | AB ( dysfunctional breathing pattern or dysfunctional breathing or breathing pattern disorder ) OR AB "breathing pattern dysfunction" OR AB "breathing dysfunction" OR AB "breathing patterns" OR AB hyperventilation OR AB ( hyperventilation syndrome or hyperventilation breathing ) OR AB "inducible laryngeal obstruction" OR AB "exercise induced laryngeal obstruction" OR AB "vocal cord dysfunction" OR AB "inspiratory stridor" OR AB "exercise induced paradoxical vocal fold motion" **Expanders** - Apply equivalent subjects **Search modes** - Boolean/Phrase | 2,129 |
|  |  | #3 | #1 and #2 | **0** |
|  | 22/05/2025 | #1 | AB "real-time feedback" OR AB biofeedback OR AB "real-time biofeedback" OR AB "real-time proprioceptive feedback" OR AB "Real-time breath training" OR AB "real-time breath analysis" OR AB "real-time breath cycle analysis" OR AB "real-time breath regulation" OR AB "real-time breathing pattern training" OR AB feedback OR AB "breathing pattern training" OR AB "breathing training" Expanders - Apply equivalent subjects Search modes - Boolean/Phrase | 99,481 |
|  |  | #2 | AB "breathing pattern re-training" OR AB "breathing re-training" Expanders - Apply equivalent subjects Search modes - Boolean/Phrase | 2 |
|  |  | #3 | "ventilation feedback training"" Expanders - Apply equivalent subjects Search modes - Boolean/Phrase | 0 |
|  |  | #4 | #1 or #2 or #3 | 99,483 |
|  |  | #5 | AB ( dysfunctional breathing pattern or dysfunctional breathing or breathing pattern disorder ) OR AB "breathing pattern dysfunction" OR AB "breathing dysfunction" OR AB "breathing patterns" OR AB hyperventilation OR AB ( hyperventilation syndrome or hyperventilation breathing ) OR AB "inducible laryngeal obstruction" OR AB "exercise induced laryngeal obstruction" OR AB "vocal cord dysfunction" OR AB "inspiratory stridor" OR AB "exercise induced paradoxical vocal fold motion" Expanders - Apply equivalent subjects Search modes - Boolean/Phrase | 2,181 |
|  |  | #6 | #4 AND #5 Limiters- Publication date 20240101-20251231 | **3** |
| 22/05/2025: 3 studies reviewed and screened out | | | |  |
| CINAHL with full text  CINAHL subject headings searched  Suggested words used | 31/01/24 | #1 | AB real-time feedback OR AB ( biofeedback or biofeedback therapy ) OR AB real-time biofeedback OR AB real-time proprioceptive feedback OR AB real-time breath training OR AB real-time breath analysis OR AB real-time breath cycle analysis OR AB real-time breath regulation OR AB real-time breathing pattern training OR AB ( feedback and performance ) OR AB feedback OR AB real-time breathing training **Expanders** - Apply equivalent subjects  **Search modes** - Boolean/Phrase | 42,676 |
|  |  | #2 | AB real-time breathing pattern training OR AB real-time breathing re-training **Expanders** - Apply equivalent subjects **Search modes** - Boolean/Phrase | 0 |
|  |  | #3 | AB ( dysfunctional breathing pattern or dysfunctional breathing or breathing pattern disorder ) OR AB breathing pattern dysfunction OR AB breathing dysfunction OR AB breathing patterns OR AB hyperventilation OR AB ( hyperventilation syndrome or hyperventilation breathing ) OR AB ( hyperventilation and treatment ) OR AB inducible laryngeal obstruction OR AB exercise induced laryngeal obstruction OR AB vocal cord dysfunction OR AB inspiratory stridor OR AB exercise induced paradoxical vocal fold motion **Expanders** - Apply equivalent subjects **Search modes** - Boolean/Phrase | 2,384 |
|  |  | #4 | S1 OR S2 **Expanders** - Apply equivalent subjects **Search modes** - Boolean/Phrase | 42,476 |
|  |  | #5 | (S1 OR S2) AND (S3 AND S4) **Expanders** - Apply equivalent subjects **Search modes** - Boolean/Phrase | **52** |
|  | 09/05/2024 | #1 | “ventilation feedback training” **Expanders** - Apply equivalent subjects **Search modes** - Boolean/Phrase | 1○ |
|  |  | #2 | AB ( dysfunctional breathing pattern or dysfunctional breathing or breathing pattern disorder ) OR AB breathing pattern dysfunction OR AB breathing dysfunction OR AB breathing patterns OR AB hyperventilation OR ( hyperventilation syndrome or hyperventilation breathing ) OR AB ( hyperventilation and treatment ) OR AB inducible laryngeal obstruction OR AB exercise induced laryngeal obstruction OR AB vocal cord dysfunction OR AB inspiratory stridor OR AB exercise induced paradoxical vocal fold motion **Expanders** - Apply equivalent subjects **Search modes** - Boolean/Phrase | 2,550 |
|  |  |  | S1 AND S2 **Expanders** - Apply equivalent subjects **Search modes** - Boolean/Phrase | **0** |
|  | 22/05/2025 | #1 | AB real-time feedback OR AB ( biofeedback or biofeedback therapy ) OR AB real-time biofeedback OR AB real-time proprioceptive feedback OR AB real-time breath training OR AB real-time breath analysis OR AB real-time breath cycle analysis OR AB real-time breath regulation OR AB real-time breathing pattern training OR AB ( feedback and performance ) OR AB feedback OR AB real-time breathing training Expanders - Apply equivalent subjects  Search modes - Boolean/Phrase | 44,515 |
|  |  | #2 | AB real-time breathing pattern training OR AB real-time breathing re-training Expanders - Apply equivalent subjects Search modes - Boolean/Phrase | 0 |
|  |  | #3 | “ventilation feedback training” Expanders - Apply equivalent subjects Search modes - Boolean/Phrase | 1 |
|  |  | #4 | S1 OR S2 OR S3 - Apply equivalent subjects Search modes - Boolean/Phrase | 44,515 |
|  |  | #5 | AB ( dysfunctional breathing pattern or dysfunctional breathing or breathing pattern disorder ) OR AB breathing pattern dysfunction OR AB breathing dysfunction OR AB breathing patterns OR AB hyperventilation OR AB ( hyperventilation syndrome or hyperventilation breathing ) OR AB ( hyperventilation and treatment ) OR AB inducible laryngeal obstruction OR AB exercise induced laryngeal obstruction OR AB vocal cord dysfunction OR AB inspiratory stridor OR AB exercise induced paradoxical vocal fold motion Expanders - Apply equivalent subjects Search modes - Boolean/Phrase | 51 |
|  |  | #6 | S4 AND S5- Apply equivalent subjects Search modes - Boolean/Phrase: Limiters- Publication Date: 20240101-20251231 | **1** |
| 09/05/2025: study already obtained; 22/05/2025: study screened and excluded | | | | |
| Cochrane Library  Word variations searched | 05/02/24 | #1 | ("real-time feedback"):ti,ab,kw OR ("biofeedback training"):ti,ab,kw OR ("real-time biofeedback"):ti,ab,kw OR ("biofeedback"):ti,ab,kw OR ("real-time proprioceptive feedback"):ti,ab,kw | 4,910 |
|  |  | #2 | ("feedback control"):ti,ab,kw OR (feedback):ti,ab,kw OR (real-time breath training):ti,ab,kw OR (real-time breathing pattern re-training):ti,ab,kw OR (real-time breathing re-training):ti,ab,kw | 24,154 |
|  |  | #3 | ("real-time breath training"):ti,ab,kw OR ("real-time breath analysis"):ti,ab,kw OR ("real-time breath cycle analysis"):ti,ab,kw OR ("real-time breath regulation"):ti,ab,kw OR ("real-time breathing pattern training"):ti,ab,kw | 2 |
|  |  | #4 | (feedback):ti,ab,kw OR (real-time breath training):ti,ab,kw OR (real-time breathing pattern re-training):ti,ab,kw OR (real-time breathing re-training):ti,ab,kw (Word variations have been searched) | 24,154 |
|  |  | #5 | **MeSH descriptor: [Feedback] explode all trees** | 1,931 |
|  |  | #6 | **MeSH descriptor: [Biofeedback, Psychology] explode all trees** | 2,140 |
|  |  | #7 | #1 OR #2 OR #3 OR #4 OR #5 OR #6 | 27,595 |
|  |  | #8 | ("dysfunctional breathing"):ti,ab,kw OR ("dysfunctional breathing patterns"):ti,ab,kw OR ("breathing pattern disorders"):ti,ab,kw OR ("breathing pattern dysfunction"):ti,ab,kw OR ("breathing dysfunction"):ti,ab,kw | 69 |
|  |  | #9 | ("breathing dysfunction"):ti,ab,kw OR ("breathing patterns"):ti,ab,kw OR ("hyperventilation"):ti,ab,kw OR ("hyperventilation syndrome"):ti,ab,kw OR ("inducible laryngeal obstruction"):ti,ab,kw | 2,197 |
|  |  | #10 | ("exercise induced laryngeal obstruction"):ti,ab,kw OR ("vocal cord dysfunction"):ti,ab,kw OR ("inspiratory stridor"):ti,ab,kw OR ("exercise induced paradoxical vocal fold motion"):ti,ab,kw | 76 |
|  |  | #11 | **MeSH descriptor: [Vocal Cord Dysfunction] explode all trees** | 106 |
|  |  | #12 | #8 OR #9 OR #10 OR #11 | 2,227 |
|  |  | #13 | #7 AND #12 | **104** |
|  | 09/05/2024 | #14 | “ventilation feedback training” |  |
|  |  | #15 | #14 AND #12 | **9** |
|  | 22/05/25 | #1 | ("real-time feedback"):ti,ab,kw OR ("biofeedback training"):ti,ab,kw OR ("real-time biofeedback"):ti,ab,kw OR ("biofeedback"):ti,ab,kw OR ("real-time proprioceptive feedback"):ti,ab,kw | 5835 |
|  |  | #2 | ("feedback control"):ti,ab,kw OR (feedback):ti,ab,kw OR (real-time breath training):ti,ab,kw OR (real-time breathing pattern re-training):ti,ab,kw OR (real-time breathing re-training):ti,ab,kw | 29230 |
|  |  | #3 | ("real-time breath training"):ti,ab,kw OR ("real-time breath analysis"):ti,ab,kw OR ("real-time breath cycle analysis"):ti,ab,kw OR ("real-time breath regulation"):ti,ab,kw OR ("real-time breathing pattern training"):ti,ab,kw | 2 |
|  |  | #4 | (feedback):ti,ab,kw OR (real-time breath training):ti,ab,kw OR (real-time breathing pattern re-training):ti,ab,kw OR (real-time breathing re-training):ti,ab,kw (Word variations have been searched) | 29230 |
|  |  | #5 | **MeSH descriptor: [Feedback] explode all trees** | 1945 |
|  |  | #6 | **MeSH descriptor: [Biofeedback, Psychology] explode all trees** | 2235 |
|  |  | #7 | #1 OR #2 OR #3 OR #4 OR #5 OR #6 | 33305 |
|  |  | #8 | ("dysfunctional breathing"):ti,ab,kw OR ("dysfunctional breathing patterns"):ti,ab,kw OR ("breathing pattern disorders"):ti,ab,kw OR ("breathing pattern dysfunction"):ti,ab,kw OR ("breathing dysfunction"):ti,ab,kw | 0 |
|  |  | #9 | ("breathing dysfunction"):ti,ab,kw OR ("breathing patterns"):ti,ab,kw OR ("hyperventilation"):ti,ab,kw OR ("hyperventilation syndrome"):ti,ab,kw OR ("inducible laryngeal obstruction"):ti,ab,kw | 1494 |
|  |  | #10 | ("exercise induced laryngeal obstruction"):ti,ab,kw OR ("vocal cord dysfunction"):ti,ab,kw OR ("inspiratory stridor"):ti,ab,kw OR ("exercise induced paradoxical vocal fold motion"):ti,ab,kw | 94 |
|  |  | #11 | **MeSH descriptor: [Vocal Cord Dysfunction] explode all trees** | 107 |
|  |  | #12 | #8 OR #9 OR #10 OR #11 | 1675 |
|  |  | #13 | #7 AND #12 | 100 |
|  |  | #14 | search limits: date published on the Cochrane Library between May 2024 and May 2025 | **8** |
| 09/05/24: study already excluded = 3; study already obtained = 1; study protocol only = 2 - excluded; already identified and awaiting screening = 1; poster = 2 – excluded  22/05/25: study reviewed and excluded = 7; Registered trial and not published = 1 | | | | |
| PEDRo  Search by PICO term | 05/02/2024 | #1 | “breathing pattern training” “breathing pattern re-training” “*feedback” Abstract and Title | **2** |
|  | 09/05/2024 | #2 | “ventilation feedback training” | **2** |
|  | 22/05/2025 | #3 | “breathing pattern training” “breathing pattern re-training” “*feedback” “ventilation feedback training”Abstract and Title | **3** |
| 09/05/2024: study already obtained = 2  22/05/2025: studies already included | | | | |
| PubMed  (Includes MEDLINE) | 30/01/24 | #1 | (((((((((((((real-time feedback[Title/Abstract]) OR (biofeedback[Title/Abstract])) OR (real-time biofeedback[Title/Abstract])) OR (real-time proprioceptive feedback[Title/Abstract])) OR (real-time breath training[Title/Abstract])) OR (real-time breath analysis[Title/Abstract])) OR (real-time breath cycle analysis[Title/Abstract])) OR (real-time breath regulation[Title/Abstract])) OR (real-time breathing pattern training[Title/Abstract])) OR (feedback[Title/Abstract])) OR (real-time breathing training[Title/Abstract])) ) OR (real-time breathing pattern re-training[Title/Abstract])) OR (real-time breathing re-training[Title/Abstract]) **Sort by:** First Author | 189,519 |
|  |  | #2 | ((((((((((((dysfunctional breathing[Title/Abstract]) OR (dysfunctional breathing patterns[Title/Abstract])) OR (breathing pattern disorders[Title/Abstract])) OR (breathing pattern dysfunction[Title/Abstract])) OR (breathing dysfunction[Title/Abstract])) OR (breathing patterns[Title/Abstract])) OR (hyperventilation[Title/Abstract])) OR (hyperventilation syndrome[Title/Abstract])) OR (inducible laryngeal obstruction[Title/Abstract])) OR (exercise induced laryngeal obstruction[Title/Abstract])) OR (vocal cord dysfunction[Title/Abstract])) OR (inspiratory stridor[Title/Abstract])) OR (exercise induced paradoxical vocal fold motion[Title/Abstract]) **Sort by:** First Author | 11,838 |
|  |  | #3 | ( "Hyperventilation/rehabilitation"[Mesh] OR "Hyperventilation/therapy"[Mesh] ) ( "Vocal Cord Dysfunction/rehabilitation"[Mesh] OR "Vocal Cord Dysfunction/therapy"[Mesh] ) **Sort by:** First Author | 1 |
|  |  | #4 | #2 OR #3 **Sort by**: First Author "dysfunctional breathing"[Title/Abstract] OR "dysfunctional breathing patterns"[Title/Abstract] OR "breathing pattern disorders"[Title/Abstract] OR "breathing pattern dysfunction"[Title/Abstract] OR "breathing dysfunction"[Title/Abstract] OR "breathing patterns"[Title/Abstract] OR "hyperventilation"[Title/Abstract] OR "hyperventilation syndrome"[Title/Abstract] OR "inducible laryngeal obstruction"[Title/Abstract] OR "exercise induced laryngeal obstruction"[Title/Abstract] OR "vocal cord dysfunction"[Title/Abstract] OR "inspiratory stridor"[Title/Abstract] OR "exercise induced paradoxical vocal fold motion"[Title/Abstract] OR (("hyperventilation/rehabilitation"[MeSH Terms] OR "hyperventilation/therapy"[MeSH Terms]) AND ("vocal cord dysfunction/rehabilitation"[MeSH Terms] OR "vocal cord dysfunction/therapy"[MeSH Terms])) | 11,838 |
|  |  | #5 | #1 AND #4 **Sort by**: First Author  ("real time feedback"[Title/Abstract] OR "biofeedback"[Title/Abstract] OR "real time biofeedback"[Title/Abstract] OR ("real-time"[All Fields] AND "proprioceptive feedback"[Title/Abstract]) OR ("real-time"[All Fields] AND "breath training"[Title/Abstract]) OR "real time breath analysis"[Title/Abstract] OR (("real-time"[All Fields] AND ("breath"[All Fields] OR "breathe"[All Fields] OR "breathed"[All Fields] OR "breathes"[All Fields] OR "breathings"[All Fields] OR "breaths"[All Fields] OR "respiration"[MeSH Terms] OR "respiration"[All Fields] OR "breathing"[All Fields])) AND "cycle analysis"[Title/Abstract]) OR ("real-time"[All Fields] AND "breath regulation"[Title/Abstract]) OR ("real-time"[All Fields] AND "breathing pattern training"[Title/Abstract]) OR "feedback"[Title/Abstract] OR ("real-time"[All Fields] AND "breathing training"[Title/Abstract]) OR (("real-time"[All Fields] AND ("breath"[All Fields] OR "breathe"[All Fields] OR "breathed"[All Fields] OR "breathes"[All Fields] OR "breathings"[All Fields] OR "breaths"[All Fields] OR "respiration"[MeSH Terms] OR "respiration"[All Fields] OR "breathing"[All Fields]) AND ("behavior"[MeSH Terms] OR "behavior"[All Fields] OR "behavioral"[All Fields] OR "behavioural"[All Fields] OR "behavior s"[All Fields] OR "behaviorally"[All Fields] OR "behaviour"[All Fields] OR "behaviourally"[All Fields] OR "behaviours"[All Fields] OR "behaviors"[All Fields] OR "pattern"[All Fields] OR "pattern s"[All Fields] OR "patternability"[All Fields] OR "patternable"[All Fields] OR "patterned"[All Fields] OR "patterning"[All Fields] OR "patternings"[All Fields] OR "patterns"[All Fields])) AND "re-training"[Title/Abstract]) OR (("real-time"[All Fields] AND ("breath"[All Fields] OR "breathe"[All Fields] OR "breathed"[All Fields] OR "breathes"[All Fields] OR "breathings"[All Fields] OR "breaths"[All Fields] OR "respiration"[MeSH Terms] OR "respiration"[All Fields] OR "breathing"[All Fields])) AND "re-training"[Title/Abstract])) AND ("dysfunctional breathing"[Title/Abstract] OR "dysfunctional breathing patterns"[Title/Abstract] OR "breathing pattern disorders"[Title/Abstract] OR "breathing pattern dysfunction"[Title/Abstract] OR "breathing dysfunction"[Title/Abstract] OR "breathing  patterns"[Title/Abstract] OR "hyperventilation"[Title/Abstract] OR "hyperventilation syndrome"[Title/Abstract] OR "inducible laryngeal obstruction"[Title/Abstract] OR "exercise induced laryngeal obstruction"[Title/Abstract] OR "vocal cord dysfunction"[Title/Abstract] OR "inspiratory stridor"[Title/Abstract] OR "exercise induced paradoxical vocal fold motion"[Title/Abstract] OR (("hyperventilation/rehabilitation"[MeSH Terms] OR "hyperventilation/therapy"[MeSH Terms]) AND ("vocal cord dysfunction/rehabilitation"[MeSH Terms] OR "vocal cord dysfunction/therapy"[MeSH Terms]))) | 174 |
|  |  | #6 | #1 AND #4 **Filters**: Humans **Sort by**: First Author  (("real time feedback"[Title/Abstract] OR "biofeedback"[Title/Abstract] OR "real time biofeedback"[Title/Abstract] OR ("real-time"[All Fields] AND "proprioceptive feedback"[Title/Abstract]) OR ("real-time"[All Fields] AND "breath training"[Title/Abstract]) OR "real time breath analysis"[Title/Abstract] OR (("real-time"[All Fields] AND ("breath"[All Fields] OR "breathe"[All Fields] OR "breathed"[All Fields] OR "breathes"[All Fields] OR "breathings"[All Fields] OR "breaths"[All Fields] OR "respiration"[MeSH Terms] OR "respiration"[All Fields] OR "breathing"[All Fields])) AND "cycle analysis"[Title/Abstract]) OR ("real-time"[All Fields] AND "breath regulation"[Title/Abstract]) OR ("real-time"[All Fields] AND "breathing pattern training"[Title/Abstract]) OR "feedback"[Title/Abstract] OR ("real-time"[All Fields] AND "breathing training"[Title/Abstract]) OR (("real-time"[All Fields] AND ("breath"[All Fields] OR "breathe"[All Fields] OR "breathed"[All Fields] OR "breathes"[All Fields] OR "breathings"[All Fields] OR "breaths"[All Fields] OR "respiration"[MeSH Terms] OR "respiration"[All Fields] OR "breathing"[All Fields]) AND ("behavior"[MeSH Terms] OR "behavior"[All Fields] OR "behavioral"[All Fields] OR "behavioural"[All Fields] OR "behavior s"[All Fields] OR "behaviorally"[All Fields] OR "behaviour"[All Fields] OR "behaviourally"[All Fields] OR "behaviours"[All Fields] OR "behaviors"[All Fields] OR "pattern"[All Fields] OR "pattern s"[All Fields] OR "patternability"[All Fields] OR "patternable"[All Fields] OR "patterned"[All Fields] OR "patterning"[All Fields] OR "patternings"[All Fields] OR "patterns"[All Fields])) AND "re-training"[Title/Abstract]) OR (("real-time"[All Fields] AND ("breath"[All Fields] OR "breathe"[All Fields] OR "breathed"[All Fields] OR "breathes"[All Fields] OR "breathings"[All Fields] OR "breaths"[All Fields] OR "respiration"[MeSH Terms] OR "respiration"[All Fields] OR "breathing"[All Fields])) AND "re-training"[Title/Abstract])) AND ("dysfunctional breathing"[Title/Abstract] OR "dysfunctional breathing patterns"[Title/Abstract] OR "breathing pattern disorders"[Title/Abstract] OR "breathing pattern dysfunction"[Title/Abstract] OR "breathing dysfunction"[Title/Abstract] OR "breathing patterns"[Title/Abstract] OR "hyperventilation"[Title/Abstract] OR "hyperventilation syndrome"[Title/Abstract] OR "inducible laryngeal obstruction"[Title/Abstract] OR "exercise induced laryngeal obstruction"[Title/Abstract] OR "vocal cord dysfunction"[Title/Abstract] OR "inspiratory stridor"[Title/Abstract] OR "exercise induced paradoxical vocal fold motion"[Title/Abstract] OR (("hyperventilation/rehabilitation"[MeSH Terms] OR "hyperventilation/therapy"[MeSH Terms]) AND ("vocal cord dysfunction/rehabilitation"[MeSH Terms] OR "vocal cord dysfunction/therapy"[MeSH Terms])))) AND (humans[Filter]) | **155** |
|  | 09/05/2024 | #1 | ("ventilation feedback training"[Title/Abstract]) AND (humans[Filter]) | **2♦** |
|  |  | #2 |  | 9,279 |
|  |  | #3 | (( "Hyperventilation/rehabilitation"[Mesh] OR "Hyperventilation/therapy"[Mesh] )) AND ( "Vocal Cord Dysfunction/rehabilitation"[Mesh] OR "Vocal Cord Dysfunction/therapy"[Mesh] ) **Sort by:** Most Recent | 1 |
|  |  | #4 | Search**:** #2 or #3 ((("dysfunctional breathing"[Title/Abstract] OR "dysfunctional breathing patterns"[Title/Abstract] OR "breathing pattern disorders"[Title/Abstract] OR "breathing pattern dysfunction"[Title/Abstract] OR "breathing dysfunction"[Title/Abstract] OR "breathing patterns"[Title/Abstract] OR "hyperventilation"[Title/Abstract] OR "hyperventilation syndrome"[Title/Abstract] OR "inducible laryngeal obstruction"[Title/Abstract] OR "exercise induced laryngeal obstruction"[Title/Abstract] OR "vocal cord dysfunction"[Title/Abstract] OR "inspiratory stridor"[Title/Abstract] OR "exercise induced paradoxical vocal fold motion"[Title/Abstract]) AND "humans"[MeSH Terms]) OR (("hyperventilation/rehabilitation"[MeSH Terms] OR "hyperventilation/therapy"[MeSH Terms]) AND ("vocal cord dysfunction/rehabilitation"[MeSH Terms] OR "vocal cord dysfunction/therapy"[MeSH Terms]))) AND (humans[Filter])  **Translations humans[Filter]:** humans[MH | 9,279 |
|  |  | #5 | #1 and #4 **Filters:** Humans ("ventilation feedback training"[Title/Abstract] AND "humans"[MeSH Terms] AND (((("dysfunctional breathing"[Title/Abstract] OR "dysfunctional breathing patterns"[Title/Abstract] OR "breathing pattern disorders"[Title/Abstract] OR "breathing pattern dysfunction"[Title/Abstract] OR "breathing dysfunction"[Title/Abstract] OR "breathing patterns"[Title/Abstract] OR "hyperventilation"[Title/Abstract] OR "hyperventilation syndrome"[Title/Abstract] OR "inducible laryngeal obstruction"[Title/Abstract] OR "exercise induced laryngeal obstruction"[Title/Abstract] OR "vocal cord dysfunction"[Title/Abstract] OR "inspiratory stridor"[Title/Abstract] OR "exercise induced paradoxical vocal fold motion"[Title/Abstract]) AND "humans"[MeSH Terms]) OR (("hyperventilation/rehabilitation"[MeSH Terms] OR "hyperventilation/therapy"[MeSH Terms]) AND ("vocal cord dysfunction/rehabilitation"[MeSH Terms] OR "vocal cord dysfunction/therapy"[MeSH Terms]))) AND "humans"[MeSH Terms])) AND (humans[Filter])  Translations humans[Filter]: humans[MH] humans[Filter]: humans[MH] humans[Filter]: humans[MH] | **0** |
|  | 22/05/2025 | #1 | Search: (((((((((((((real-time feedback[Title/Abstract]) OR (biofeedback[Title/Abstract])) OR (real-time biofeedback[Title/Abstract])) OR (real-time proprioceptive feedback[Title/Abstract])) OR (real-time breath training[Title/Abstract])) OR (real-time breath analysis[Title/Abstract])) OR (real-time breath cycle analysis[Title/Abstract])) OR (real-time breath regulation[Title/Abstract])) OR (real-time breathing pattern training[Title/Abstract])) OR (feedback[Title/Abstract])) OR (real-time breathing training[Title/Abstract])) ) OR (real-time breathing pattern re-training[Title/Abstract])) OR (real-time breathing re-training[Title/Abstract]) | 208,784 |
|  |  | #2 | Search: ((((((((((((dysfunctional breathing[Title/Abstract]) OR (dysfunctional breathing patterns[Title/Abstract])) OR (breathing pattern disorders[Title/Abstract])) OR (breathing pattern dysfunction[Title/Abstract])) OR (breathing dysfunction[Title/Abstract])) OR (breathing patterns[Title/Abstract])) OR (hyperventilation[Title/Abstract])) OR (hyperventilation syndrome[Title/Abstract])) OR (inducible laryngeal obstruction[Title/Abstract])) OR (exercise induced laryngeal obstruction[Title/Abstract])) OR (vocal cord dysfunction[Title/Abstract])) OR (inspiratory stridor[Title/Abstract])) OR (exercise induced paradoxical vocal fold motion[Title/Abstract]) | 12, 296 |
|  |  | #3 | Search: ( "Hyperventilation/rehabilitation"[Mesh] OR "Hyperventilation/therapy"[Mesh] ) ( "Vocal Cord Dysfunction/rehabilitation"[Mesh] OR "Vocal Cord Dysfunction/therapy"[Mesh] ) | 1 |
|  |  | #4 | Search: "ventilation feedback training" | 2 |
|  |  | #5 | Search: #2 or #3 | 12, 296 |
|  |  | #6 | Search: #1 or #4 | 208,784 |
|  |  | #7 | Search: #5 AND #6 | 187 |
|  |  | #8 | Search: #5 AND #6 Filters: from 2024/5/1 - 2025/5/22 | **12** |
| 09/05/2024:♦both of these studies already screened  22/05/2025: 12 studies reviewed and screened out | | | | |
| Scopus | 05/02/24 | #1 | ( ABS ( "real-time feedback" ) OR ABS ( biofeedback ) OR ABS ( "real-time biofeedback" ) OR ABS ( "real-time proprioceptive feedback" ) OR ABS ( "real-time breath training" ) OR ABS ( "real-time breath analysis" ) OR ABS ( "real-time breath cycle analysis" ) OR ABS ( "real-time breath regulation" ) OR ABS ( "real-time breathing pattern training" ) OR ABS ( feedback ) OR ABS ( "real-time breathing training" ) OR ABS ( "real-time breathing pattern re-training" ) OR ABS ( "real-time breathing re-training" ) ) | 701,312 |
|  |  | #2 | ( ABS ( "dysfunctional breathing" ) OR ABS ( "dysfunctional breathing patterns" ) OR ABS ( "breathing pattern disorders" ) OR ABS ( "breathing pattern dysfunction" ) OR ABS ( "breathing dysfunction" ) OR ABS ( "breathing patterns" ) OR ABS ( "hyperventilation" ) OR ABS ( "hyperventilation syndrome" ) OR ABS ( "inducible laryngeal obstruction" ) OR ABS ( "exercise induced laryngeal obstruction" ) OR ABS ( "vocal cord dysfunction" ) OR ABS ( "inspiratory stridor" ) OR ABS ( "exercise induced paradoxical vocal fold motion" ) ) | 16,296 |
|  |  | #3 | ( ( ABS ( "real-time feedback" ) OR ABS ( biofeedback ) OR ABS ( "real-time biofeedback" ) OR ABS ( "real-time proprioceptive feedback" ) OR ABS ( "real-time breath training" ) OR ABS ( "real-time breath analysis" ) OR ABS ( "real-time breath cycle analysis" ) OR ABS ( "real-time breath regulation" ) OR ABS ( "real-time breathing pattern training" ) OR ABS ( feedback ) OR ABS ( "real-time breathing training" ) OR ABS ( "real-time breathing pattern re-training" ) OR ABS ( "real-time breathing re-training" ) ) ) AND ( ( ABS ( "dysfunctional breathing" ) OR ABS ( "dysfunctional breathing patterns" ) OR ABS ( "breathing pattern disorders" ) OR ABS ( "breathing pattern dysfunction" ) OR ABS ( "breathing dysfunction" ) OR ABS ( "breathing patterns" ) OR ABS ( "hyperventilation" ) OR ABS ( "hyperventilation syndrome" ) OR ABS ( "inducible laryngeal obstruction" ) OR ABS ( "exercise induced laryngeal obstruction" ) OR ABS ( "vocal cord dysfunction" ) OR ABS ( "inspiratory stridor" ) OR ABS ( "exercise induced paradoxical vocal fold motion" ) ) ) | 334 |
|  |  | #4 | ( ( ABS ( "real-time feedback" ) OR ABS ( biofeedback ) OR ABS ( "real-time biofeedback" ) OR ABS ( "real-time proprioceptive feedback" ) OR ABS ( "real-time breath training" ) OR ABS ( "real-time breath analysis" ) OR ABS ( "real-time breath cycle analysis" ) OR ABS ( "real-time breath regulation" ) OR ABS ( "real-time breathing pattern training" ) OR ABS ( feedback ) OR ABS ( "real-time breathing training" ) OR ABS ( "real-time breathing pattern re-training" ) OR ABS ( "real-time breathing re-training" ) ) ) AND ( ( ABS ( "dysfunctional breathing" ) OR ABS ( "dysfunctional breathing patterns" ) OR ABS ( "breathing pattern disorders" ) OR ABS ( "breathing pattern dysfunction" ) OR ABS ( "breathing dysfunction" ) OR ABS ( "breathing patterns" ) OR ABS ( "hyperventilation" ) OR ABS ( "hyperventilation syndrome" ) OR ABS ( "inducible laryngeal obstruction" ) OR ABS ( "exercise induced laryngeal obstruction" ) OR ABS ( "vocal cord dysfunction" ) OR ABS ( "inspiratory stridor" ) OR ABS ( "exercise induced paradoxical vocal fold motion" ) ) ) **AND ( EXCLUDE ( EXACTKEYWORD , "nonhuman" ) OR EXCLUDE ( EXACTKEYWORD , "animal experiment" ) OR EXCLUDE ( EXACTKEYWORD , "animal" ) OR EXCLUDE ( EXACTKEYWORD , "animals" ) )** | **310** |
| Initial search with title/abstract/keyword returned a large amount of irrelevant studies. Therefore repeated using Abstract and returned 334. Keyword excluded: non-human, animal experiment, animal, animals. Returned 281 | | | | |
|  | 09/05/2024 | #1 | ABS ( "ventilation feedback training" ) | **1** |
|  |  | #2 | ( ABS ( "ventilation feedback training" ) AND ABS ( "dysfunctional breathing" ) OR ABS ( "dysfunctional breathing patterns" ) OR ABS ( "breathing pattern disorders" ) OR ABS ( "breathing pattern dysfunction" ) OR ABS ( "breathing dysfunction" ) OR ABS ( "breathing patterns" ) OR ABS ( "hyperventilation" ) OR ABS ( "hyperventilation syndrome" ) OR ABS ( "inducible laryngeal obstruction" ) OR ABS ( "exercise induced laryngeal obstruction" ) OR ABS ( "vocal cord dysfunction" ) OR ABS ( "inspiratory stridor" ) OR ABS ( "exercise induced paradoxical vocal fold motion" ) ) | 0 |
|  | 22/05/2025 | #1 | ( ABS ( "real-time feedback" ) OR ABS ( biofeedback ) OR ABS ( "real-time biofeedback" ) OR ABS ( "real-time proprioceptive feedback" ) OR ABS ( "real-time breath training" ) OR ABS ( "real-time breath analysis" ) OR ABS ( "real-time breath cycle analysis" ) OR ABS ( "real-time breath regulation" ) OR ABS ( "real-time breathing pattern training" ) OR ABS ( feedback ) OR ABS ( "real-time breathing training" ) OR ABS ( "real-time breathing pattern re-training" ) OR ABS ( "real-time breathing re-training" ) ) | 772,348 |
|  |  | #2 | ( ABS ( "dysfunctional breathing" ) OR ABS ( "dysfunctional breathing patterns" ) OR ABS ( "breathing pattern disorders" ) OR ABS ( "breathing pattern dysfunction" ) OR ABS ( "breathing dysfunction" ) OR ABS ( "breathing patterns" ) OR ABS ( "hyperventilation" ) OR ABS ( "hyperventilation syndrome" ) OR ABS ( "inducible laryngeal obstruction" ) OR ABS ( "exercise induced laryngeal obstruction" ) OR ABS ( "vocal cord dysfunction" ) OR ABS ( "inspiratory stridor" ) OR ABS ( "exercise induced paradoxical vocal fold motion" ) ) | 17, 044 |
|  |  | #3 | ABS ( "ventilation feedback training" ) | 1 |
|  |  | #4 | ( ( ABS ( "real-time feedback" ) OR ABS ( biofeedback ) OR ABS ( "real-time biofeedback" ) OR ABS ( "real-time proprioceptive feedback" ) OR ABS ( "real-time breath training" ) OR ABS ( "real-time breath analysis" ) OR ABS ( "real-time breath cycle analysis" ) OR ABS ( "real-time breath regulation" ) OR ABS ( "real-time breathing pattern training" ) OR ABS ( feedback ) OR ABS ( "real-time breathing training" ) OR ABS ( "real-time breathing pattern re-training" ) OR ABS ( "real-time breathing re-training" ) ) ) OR ( ABS ( "ventilation feedback training" ) ) | 772, 348 |
|  |  | #5 | ( ( ABS ( "real-time feedback" ) OR ABS ( biofeedback ) OR ABS ( "real-time biofeedback" ) OR ABS ( "real-time proprioceptive feedback" ) OR ABS ( "real-time breath training" ) OR ABS ( "real-time breath analysis" ) OR ABS ( "real-time breath cycle analysis" ) OR ABS ( "real-time breath regulation" ) OR ABS ( "real-time breathing pattern training" ) OR ABS ( feedback ) OR ABS ( "real-time breathing training" ) OR ABS ( "real-time breathing pattern re-training" ) OR ABS ( "real-time breathing re-training" ) ) ) OR ( ABS ( "ventilation feedback training" ) ) ) AND ( ( ABS ( "dysfunctional breathing" ) OR ABS ( "dysfunctional breathing patterns" ) OR ABS ( "breathing pattern disorders" ) OR ABS ( "breathing pattern dysfunction" ) OR ABS ( "breathing dysfunction" ) OR ABS ( "breathing patterns" ) OR ABS ( "hyperventilation" ) OR ABS ( "hyperventilation syndrome" ) OR ABS ( "inducible laryngeal obstruction" ) OR ABS ( "exercise induced laryngeal obstruction" ) OR ABS ( "vocal cord dysfunction" ) OR ABS ( "inspiratory stridor" ) OR ABS ( "exercise induced paradoxical vocal fold motion" ) ) ) | 363 |
|  |  | #6 | ( ( ( ABS ( "real-time feedback" ) OR ABS ( biofeedback ) OR ABS ( "real-time biofeedback" ) OR ABS ( "real-time proprioceptive feedback" ) OR ABS ( "real-time breath training" ) OR ABS ( "real-time breath analysis" ) OR ABS ( "real-time breath cycle analysis" ) OR ABS ( "real-time breath regulation" ) OR ABS ( "real-time breathing pattern training" ) OR ABS ( feedback ) OR ABS ( "real-time breathing training" ) OR ABS ( "real-time breathing pattern re-training" ) OR ABS ( "real-time breathing re-training" ) ) ) OR ( ABS ( "ventilation feedback training" ) ) ) AND ( ( ABS ( "dysfunctional breathing" ) OR ABS ( "dysfunctional breathing patterns" ) OR ABS ( "breathing pattern disorders" ) OR ABS ( "breathing pattern dysfunction" ) OR ABS ( "breathing dysfunction" ) OR ABS ( "breathing patterns" ) OR ABS ( "hyperventilation" ) OR ABS ( "hyperventilation syndrome" ) OR ABS ( "inducible laryngeal obstruction" ) OR ABS ( "exercise induced laryngeal obstruction" ) OR ABS ( "vocal cord dysfunction" ) OR ABS ( "inspiratory stridor" ) OR ABS ( "exercise induced paradoxical vocal fold motion" ) ) ) AND PUBYEAR > 2023 AND PUBYEAR < 2026 | **29** |
| 09/05/2024: study already included  22/05/2025: 29 articles reviewed and screened out | | | | |
| Web of Science | 05/02/2024 | #1 | ((((((((((((ALL=("real-time feedback")) OR ALL=(biofeedback)) OR ALL=("real-time biofeedback")) OR ALL=("real-time proprioceptive feedback")) OR ALL=("real-time breath training")) OR ALL=("real-time breath analysis")) OR ALL=("real-time breath cycle analysis")) OR ALL=("real-time breath regulation")) OR ALL=("real-time breathing pattern training")) OR ALL=(feedback)) OR ALL=("real-time breathing training")) OR ALL=("real-time breathing pattern re-training")) OR ALL=("real-time breathing pattern re-training") | 710,760 |
|  |  | #2 | ((((((((((((ALL=("dysfunctional breathing")) OR ALL=("dysfunctional breathing patterns")) OR ALL=("breathing pattern disorders")) OR ALL=("breathing pattern dysfunction")) OR ALL=("breathing dysfunction")) OR ALL=("breathing patterns")) OR ALL=(hyperventilation)) OR ALL=("hyperventilation syndrome")) OR ALL=("inducible laryngeal obstruction")) OR ALL=("exercise induced laryngeal obstruction")) OR ALL=("vocal cord dysfunction")) OR ALL=("inspiratory stridor")) OR ALL=("exercise induced paradoxical vocal fold motion") | 11,987 |
|  |  | #3 | #1 AND #2 | **283** |
|  | 09/05/2024 | #1 | "ventilation feedback training" (All Fields) | 2♠ |
|  |  | #2 | ((((((((((((ALL=("dysfunctional breathing")) OR ALL=("dysfunctional breathing patterns")) OR ALL=("breathing pattern disorders")) OR ALL=("breathing pattern dysfunction")) OR ALL=("breathing dysfunction")) OR ALL=("breathing patterns")) OR ALL=(hyperventilation)) OR ALL=("hyperventilation syndrome")) OR ALL=("inducible laryngeal obstruction")) OR ALL=("exercise induced laryngeal obstruction")) OR ALL=("vocal cord dysfunction")) OR ALL=("inspiratory stridor")) OR ALL=("exercise induced paradoxical vocal fold motion") | 12,471 |
|  |  | #3 | #1 AND #2 | **0** |
|  | 22/05/2025 | #1 | ALL=("real-time feedback" OR "biofeedback" OR "real-time biofeedback" OR "real-time proprioceptive feedback" OR "real-time breath training" OR "real-time breath analysis" OR "real-time breath cycle analysis" OR "real-time breath regulation" OR "real-time pattern training" OR "feedback" OR "real-time breathing training" OR "real-time breathing pattern re-training" OR "real-time breathing pattern re-training" OR "ventilation feedback training") | 817,609 |
|  |  | #2 | ALL=("dysfunctional breathing" OR "dysfunctional breathing patterns" OR "breathing pattern disorders" OR "breathing pattern dysfunction" OR "breathing dysfunction" OR "breathing patterns" OR "hyperventilation" OR "hyperventilation syndrome" OR "inducible laryngeal obstruction" OR "exercise induced laryngeal obstruction" OR "vocal cord dysfunction" OR "inspiratory stridor" OR "exercise induced paradoxical vocal fold motion") | 13,793 |
|  |  | #3 | #2 AND #1 | 301 |
|  |  | #4 | #2 AND #1 and 2024 or 2025 (Publication Years) | **17** |
| 09/05/2024: 2 studies already included  22/05/2025: 17 articles reviewed and screened out | | | | |
| Registry | Date of Search | Search Number | **Search Terms** | Results |
| ClinicalTrials.gov  [www.clinicaltrials.gov](http://www.clinicaltrials.gov) | 06/02/24 | #1 | **Condition or disease:** "dysfunctional breathing" or "dysfunctional breathing patterns" or "breathing pattern disorders" or "breathing pattern dysfunction" or "breathing dysfunction" or "breathing patterns" or hyperventilation or "hyperventilation syndrome" or "inducible laryngeal obstruction" or "exercise induced laryngeal obstruction" or "vocal cord dysfunction" or "inspiratory stridor" or "exercise induced paradoxical vocal fold motion"  **Other terms:** "real-time feedback" or biofeedback or "real-time biofeedback" or "real-time proprioceptive feedback" or "real-time breath training" or "real-time breath analysis" or "rea-time breath cycle analysis" or "real-time breath regulation" or "real-time breathing pattern training" or "feedback" or "real-time breathing training" or "real-time breathing pattern re-training" or "real-time breathing re-training"  **Intervention/treatment:** "breathing pattern training" or "breathing pattern re-training"  **Other terms:** biofeedback | **1♣** |
|  | ♣11 studies identified but 4 actively recruiting; No results published or posted for 6 | | | |
|  | 09/05/2024 | #1 | **Condition or disease:** "dysfunctional breathing" or "dysfunctional breathing patterns" or "breathing pattern disorders" or "breathing pattern dysfunction" or "breathing dysfunction" or "breathing patterns" or hyperventilation or "hyperventilation syndrome" or "inducible laryngeal obstruction" or "exercise induced laryngeal obstruction" or "vocal cord dysfunction" or "inspiratory stridor" or "exercise induced paradoxical vocal fold motion"  **Other terms:** biofeedback or “biofeedback training”  **Intervention/treatment:** "ventilation feedback training" | **0** |
|  | 22/05/2025 |  | **Condition/disease**: "dysfunctional breathing" or "dysfunctional breathing patterns" or "breathing pattern disorders" or "breathing pattern dysfunction" or "breathing dysfunction" or "breathing patterns" or hyperventilation or "hyperventilation syndrome" or "inducible laryngeal obstruction" or "exercise induced laryngeal obstruction" or "vocal cord dysfunction" or "inspiratory stridor" or "exercise induced paradoxical vocal fold motion"  **Other terms:** "real-time feedback" or biofeedback or "real-time biofeedback" or "real-time proprioceptive feedback" or "real-time breath training" or "real-time breath analysis" or "rea-time breath cycle analysis" or "real-time breath regulation" or "real-time breathing pattern training" or "feedback" or "real-time breathing training" or "real-time breathing pattern re-training" or "real-time breathing re-training" or "ventilation feedback training"  **Intervention/treatment:** "breathing pattern training" or "breathing pattern re-training" | **0** |
| ICTRP  [www.trialsearch.who.int](http://www.trialsearch.who.int) | 06/02/2014 | #1 | **Title**: breathing pattern training  **Condition:** "dysfunctional breathing" or "dysfunctional breathing patterns" or "breathing pattern disorders" or "breathing pattern dysfunction" or "breathing dysfunction" or "breathing patterns" or hyperventilation or "hyperventilation syndrome" or "inducible laryngeal obstruction" or "exercise induced laryngeal obstruction" or "vocal cord dysfunction" or "inspiratory stridor" or "exercise induced paradoxical vocal fold motion"  **Intervention:** "real-time feedback" or biofeedback or "real-time biofeedback" or "real-time proprioceptive feedback" or "real-time breath training" or "real-time breath analysis" or "rea-time breath cycle analysis" or "real-time breath regulation" or "real-time breathing pattern training" or "feedback" or "real-time breathing training" or "real-time breathing pattern re-training" or "real-time breathing re-training"  **Condition:** breathing pattern disorder or dysfunctional breathing  **Intervention:** Biofeedback | **1** |
|  | 09/05/2024 | #1 | **Title:** ventilation feedback training  **Condition**: "dysfunctional breathing" or "dysfunctional breathing patterns" or "breathing pattern disorders" or "breathing pattern dysfunction" or "breathing dysfunction" or "breathing patterns" or hyperventilation or "hyperventilation syndrome" or "inducible laryngeal obstruction" or "exercise induced laryngeal obstruction" or "vocal cord dysfunction" or "inspiratory stridor" or "exercise induced paradoxical vocal fold motion"  **Intervention**: "real-time feedback" or biofeedback or "real-time biofeedback" or "real-time proprioceptive feedback" or "real-time breath training" or "real-time breath analysis" or "rea-time breath cycle analysis" or "real-time breath regulation" or "real-time breathing pattern training" or "feedback" or "real-time breathing training" or "real-time breathing pattern re-training" or "real-time breathing re-training" | **0** |
|  | 22/05/25 | #1 | Title: breathing pattern training  **Condition:** "dysfunctional breathing" or "dysfunctional breathing patterns" or "breathing pattern disorders" or "breathing pattern dysfunction" or "breathing dysfunction" or "breathing patterns" or hyperventilation or "hyperventilation syndrome" or "inducible laryngeal obstruction" or "exercise induced laryngeal obstruction" or "vocal cord dysfunction" or "inspiratory stridor" or "exercise induced paradoxical vocal fold motion"  **Intervention:** "real-time feedback" or biofeedback or "real-time biofeedback" or "real-time proprioceptive feedback" or "real-time breath training" or "real-time breath analysis" or "rea-time breath cycle analysis" or "real-time breath regulation" or "real-time breathing pattern training" or "feedback" or "real-time breathing training" or "real-time breathing pattern re-training" or "real-time breathing re-training" or “ventilation feedback training”  **Condition:** breathing pattern disorder or dysfunctional breathing  Intervention: Biofeedback | **0** |
| **Archive** | **Date of Search** | **Search Number** | **Search Terms** | **Results** |
| BioRxiv and MedRxiv | 06/02/24 | #1 | “breathing pattern training” or “breathing training” AND biofeedback or feedback | **0** |
|  |  | #2 | “breathing pattern re-training” or “breathing re-training” AND biofeedback or feedback | **49** |
|  | 09/05/24 | #3 | “ventilation feedback training” | **0** |
|  | 22/05/2025 | #1 | “breathing pattern training” or “breathing training” AND biofeedback or feedback: Refined 01 May 2024 and 22 may 2025 | **49** |
|  |  | #2 | “breathing pattern re-training” or “breathing re-training” AND biofeedback or feedback Refined 01 May 2024 and 22 may 2025 | **0** |
|  |  | #3 | “ventilation feedback training” Refined 01 May 2024 and 22 may 2025 | **0** |
| 22/05/2025: no new journals | | | | |
