# Supplementary material for: Biofeedback in Breathing Pattern Training: A Systematic Review
Source: Can Respir J. 2026 Nov 15;2026:5567144. doi: 10.1155/carj/5567144 (PMC13770288; doi:10.1155/carj/5567144)
Supplement: Supplementary file 2 [file CARJ-2026-5567144-s002.docx]

| **Database** | **Platform/Provider** | **Coverage** |
| --- | --- | --- |
|  |  |  |
| APA PsycInfo  SPORTDiscus | EBSCOhost | 1806 – present  1892 - present |
| CINAHL Plus with full text | EBSCOhost | 1961 - present |
| Cochrane Library | Wiley Online Library | 1996 - present |
| PEDRo | PEDRo Partnership | 1929 - present |
| PubMed | United States National  Library of Medicine | 1946 - present |
| Scopus | Elsevier | 1788 - present |
| Web of Science | Clarivate | 1900 - present |
| **Registry** |  |  |
| ClinicalTrials.gov  [www.clinicaltrials.gov](http://www.clinicaltrials.gov) | United States National Library  of Medicine | 2000 - present |
| International Clinical Trials  Registry Platform  <https://trialsearch.who.int> | World Health organisation | 2005 - present |
| **Archive** |  |  |
| BioRxiv | Cold Spring Harbor Laboratory | 2013 - present |
| MedRxiv | Cold Spring Harbor Laboratory | 2019 - present |

**Supplementary Material 3. Summary of information sources**

Relevant grey literature identified from the information sources were considered. The reference lists of all included studies were reviewed for any additional and relevant studies. The systematic review team also searched their personal files for any additional and relevant studies. If studies were presented in languages other than English, they were included if an adequate translation was possible using Google Translate. Adequacy was determined by consensus agreement. Study reviewers were not blind to journal title, date of publication, authors, or institutions. Two study authors were contacted for additional information to inform decision making.
