# Supplementary material for: Biofeedback in Breathing Pattern Training: A Systematic Review
Source: Can Respir J. 2026 Nov 15;2026:5567144. doi: 10.1155/carj/5567144 (PMC13770288; doi:10.1155/carj/5567144)
Supplement: Supplementary file 4 [file CARJ-2026-5567144-s004.docx]

| **Study** | **Supplementary Material 5: Additional Information for Protocols** |
| --- | --- |
| Ishii *et al,* 2021 | **General:** Respiratory parameters measured breath by breath using an expired gas analyser. A specially designed rebreathing circuit was made to create hyperventilation by rebreathing. Ventilation volume set to match that of moderate exercise intensity by monitoring the concentrations of oxygen and carbon dioxide. Subjects supported in a wheelchair reclined to 60º. Head, upper and lower limbs supported. Rebreathing load set to a minute ventilation equivalent to moderate exercise intensity. The values for RatioTH-ABD and ventilatory parameters calculated as the average value of three minutes. Breathing pattern analysed using inductance plethysmography. ECG and SpO_2_ also monitored. **Biofeedback:** Analog signals obtained by the expired gas analyser and inductance plethysmography converted to digital format. **Exercise:** No exercise component to this study. |
| Smyth *et al,* 2021 | **General:** Both groups received breathing training instructions focussing on “using diaphragm and intercostal muscles to initiate the movement of the breath, move the lower ribcage laterally, avoid initiating breath from shoulders and abdomen, move the ribcage and abdomen together and aim for a smooth inhalation using the real-time feedback as an aid”. **Biofeedback:** Participants given time to practice implementing the feedback using the real-time feedback system for as long as required, typically for approximately thirty minutes. **Exercise:** Participants performed a sub-maximal exercise challenge positioned upright on the cycle ergometer with arms extended to the sides and rested on stands. Cycle test began at 50W and increased by 30W every minute. Exercise intensity defined using rate of perceived exertion values of 11, 13-14, and 17-18 representing low, moderate and high exercise intensity. |
| Van Gestel *et al*, 2012 | **General:** All participants allocated to cardiopulmonary training in an outpatient clinic 3 times per week (1.5-hour sessions) for 3-4 weeks. Totalling 10 sessions. Sessions included dynamic strength training (quadriceps femoris, hamstrings, triceps surae, pectoralis major, deltoid, latissimus dorsi and triceps brachii). Exercises performed in sitting starting at 70% of 1-repitition maximum and completed 3 cycles of 10 repetitions of isotonic contractions with a rest of 2 minutes between the series. When patients were able to perform 3 sets of 10 repetitions without any difficulty, effort was increased stepwise by 5% of the 1 repetition maximum. Cardiopulmonary endurance training was performed on a cycle ergometer, using stepping exercises and arm cranking. Initial intensity of cycling set at 30% peak workload for 20 minutes and increased according to symptom scores. **Biofeedback:** 10 sessions of 30 minutes of controlled breathing. Instruction given during first session. The respiratory biofeedback system visually displayed the desired respiratory pattern. Patients were trained to improve their breathing pattern at rest for 10 minutes while seated and instructed to do this daily. Patients instructed to modify breathing characteristics (rapid shallow breathing, breath-to-breath irregularity in rate and depth and predominant thoracic breathing). Breathing pattern monitored by respiratory sensors at umbilical and abdominal level and signals converted to visual and acoustic outputs. Signals used simultaneously via earphone and overhead screen. **Exercise:** The intervention group were trained to improve their breathing during 20 minutes of cardiopulmonary endurance training on a cycle ergometer. |
| Collins *et al,* 2008 | **General/biofeedback:** Ventilation feedback alone; Goals and parameters for expiratory flow rate, Te:Ti and RR were determined from the results of a pilot study and from patient baseline measurements. Expiratory flow rate initially set at 0.12 L/s, Te:Ti ratio was set at 95% of the ratio recorded during the resting period at baseline assessment and RR set at 1-2 breaths less than baseline RR. When the patient could hit the inhalation/exhalation targets >85% of the time during a 5-minute bout of training the Te: Ti ratio, RR and minimum expiratory flow rate were progressively changed. Te:Ti changed to a value 12% greater than baseline, RR 1-2 breaths less than the RR recorded at 60% of VO_2_ peak recorded at baseline and minimum expiratory flow increased to 0.14 L/s. Once these setting reached, they were not adjusted any further but were titrated down if required until the patient could hit at least 85% of targets. Ventilation feedback plus exercise; Protocol as above but timings as for exercise protocol. **Exercise:** Baseline exercise testing consisted of a leg cycle and treadmill symptom limited test and a treadmill constant work rate test. The exercise alone group exercised over thirty-six sessions (eighteen of leg cycle exercise, followed by eighteen of treadmill exercise). Baseline testing repeated after the first eighteen sessions with additional leg cycle constant work rate test. Upper body resistance training at least twice a week. Patient choice of TheraBand and/or lifting light dumb bells. One set of 12 reps progressing to two sets of 12 reps. If two sets were completed during three consecutive sessions, then weight increased by 2-3 pounds or TheraBand thickness increased. Exercise intensity set as a percentage of baseline peak oxygen uptake. Starting at 60% and progressively increased to 85%. Training lasted 25-45 minutes, three times a week. Post-study testing with treadmill symptom limited test and treadmill constant work rate test. Ventilation feedback plus exercise; Exercise protocol as above. Ventilation feedback alone; 30-35 minutes including 10 minutes of low intensity activity (eighteen sessions of unloaded cycling and eighteen sessions of treadmill at 1.8mph at 0% grade). |
| Collins *et al,* 2003 | **General:** For ventilation feedback only and ventilation-feedback with exercise; The ventilation feedback system enabled bidirectional airflow measurement. Subjects breathed through a mouthpiece. **Biofeedback:** Subjects received visual feedback via a computer monitor. The feedback screen was divided in half. Left side for inhalation, right side for exhalation. If the expiratory phase continued for the requisite length of time the subject scored a hit. If expiration was interrupted a miss was recorded. Each hit was accompanied by an audible tone. Tally of hits and misses was presented on the screen. A performance score was computed at the end of each specified training interval. The duration of Te required to score a hit was specified by the investigator as a multiple of Ti. Te was set at 10% above that recorded at any given workload (%VO_2_). Each new target Te was computed from the immediately preceding inspiration or a rolling average of previous Ti. The investigator could also get a minimal threshold expiratory flow parameter. Each session included a 3 min ventilation feedback practice without exercise (ventilation feedback groups only) and a 3-5 min warm up, interval training and cool- down. **Exercise:** Prior to randomisation, patients completed a symptom-limited leg-cycle ergometer test and two constant work-rate leg-cycle ergometer tests at a workload equal to 85% of the maximal power output of the symptom limited cycle test. All exercise tests separated by a t least 48 hours. If the difference in exercise duration was >10%, a third test was completed. Following randomisation, individualized interval training prescription given to each patient. 65-75% of each training session included exercise at an intensity of 80-85% of peak oxygen intake (VO_2_peak). Every training session included one or two sets of progressive resistance upper body exercise using elastic bands or dumb bells. All subjects expected to exercise for approx. 30-50 minutes, three times a week. Exercise only and ventilation feedback and exercise groups trained at the same intensity. Ventilation feedback only trained with minimal physical activity i.e. 0W workload during cycling. Limited to 10 minutes only and thus not of sufficient intensity to provide a training effect. After the leg cycle subjects completed an upper body muscle endurance/strength training session, using TheraBand and dumb bells. This consisted of 6-8 exercises. One to two sets with reps of 12 to 15. Constant work-rate leg cycle ergometer test performed at 85% of maximal power at baseline and at six weeks. |
| Estève *et al,* 1996 | **General:** Subject seated facing computer screen. Breathing then recorded for 30 seconds. **Biofeedback:** Longest and largest amplitude breath from this recording used as the target breath with a ±10-15% envelope. Each session consisted of seven sets of 10 breaths with feedback followed by seven sets of 5 breaths without feedback. During no feedback runs, breath was not displayed, and subject asked to maintain the same breathing pattern as during feedback. Target adjusted to patients’ skill and progress. During training target breath and target displayed. BPT session lasted 20 mins. Planned for twice a day for two weeks and once a day for two weeks. FEV_1_ and FVC measured on arrival and leaving the rehabilitation centre. **Exercise:** No exercise component to this study. |
| Sackner *et al,* 1984 | General: Breath by breath analysis with RIP and pre and post validation with spirometry. **Exercise:** No exercise component to this study |
| Van Doorn *et al,* 1982 | **General:** Biofeedback group; Short interviews pre-intervention. Baseline end-tidal PC0_2_ established. **Biofeedback:** Thirty-minute PCO_2_ feedback split up in two-minute trials with a pause after every third trial. Feedback showed actual end tidal PCO_2_ and a desired level set at 1.5mmHg above patients end-tidal PCO_2_ level. Patients seated and breathed through a facemask. Patients instructed to ensure peak PCO_2_ measurements hit desired levels. When a trial was successfully completed (80% of hits) visual feedback changed to binary auditory feedback. A block of two successful trails was supplemented with a trial without feedback. Then desired PCO_2_ level raised another 1.5mmHg and same procedure repeated. A maximum of seven weekly sessions. Sessions stopped when patients were able to maintain PCO_2_ >37.5mmHg in one session. After the second session patients were advised to practice the learned skills at home. Breathing group: subjects given a training schedule of breathing instructions to be used at home twice a day for 10 minutes (slow and shallow abdominal breathing), counting RR. Four meetings within the seven weeks were arranged so that the subject could discuss progress with therapist. Seven minutes adaptation time before each session. The frequency of HVS symptoms recorded for a two-week period at pre and post treatment and follow-up evaluation. **Exercise:** No exercise component to this study. |

**Definitions of abbreviations:** BPT = breathing pattern training; ECG = electrocardiogram; FEV_1_ = forced expiratory volume in 1 second; FVC = forced vital capacity; HVS =.hyperventilation syndrome; mmHg = millimetres of mercury; mph = miles per hour; PCO_2_ = partial pressure of carbon dioxide; RatioTH-ABD = ratio of thoracoabdominal contribution to tidal volume; RIP = respiratory inductive plethysmography; RR = respiratory rate; SpO_2_ = transcutaneous oxygen saturation; Te = expiratory time; Ti = inspiratory time; VO_2_peak = Peak oxygen consumption; W = Watts; L/s = Litres/second.
