# Supplementary material for: Biofeedback in Breathing Pattern Training: A Systematic Review
Source: Can Respir J. 2026 Nov 15;2026:5567144. doi: 10.1155/carj/5567144 (PMC13770288; doi:10.1155/carj/5567144)
Supplement: Supplementary file 5 [file CARJ-2026-5567144-s005.docx]

**Supplementary Material 6. Risk of bias per outcome.**

RoB 2: Risk of Bias summaries by outcome:

RoB 2 considers the risk of bias in any type of randomised trial using five domains to identify an overall risk of bias judgement. Risk is defined as low risk of bias, some concerns and high risk of bias.

 **Low** **Some concerns** **High	○ Outcome not measured**

**Domains: D1: Bias arising from the randomisation process; D2: Bias due to deviations from intended intervention; D3: Bias due to missing outcome data; D4: Bias in measurement of the outcome; D5: Bias in selection of the reported result.** Definition of abbreviations: TAM = thoracoabdominal movement; CWS = chest wall synchrony; HVS = Hyperventilation syndrome; FEV_1_ = forced expiratory volume in 1 second; FVC = forced vital capacity; PCO_2_ = partial pressure of carbon dioxide; RoB 2 = Cochrane risk of bias tool for randomised studies; ROBINS I = Cochrane risk of bias tool in non-randomised studies of interventions.

| **Outcome:**  **TAM/CWS** | D1 | D2 | D3 | D4 | D5 | Overall |
| --- | --- | --- | --- | --- | --- | --- |
| Smyth et al, 2021 |  |  |  |  |  |  |
| van Gestel et al, 2012 | ○ | ○ | ○ | ○ | ○ | ○ |
| Collins et al, 2008 | ○ | ○ | ○ | ○ | ○ | ○ |
| Collins et al, 2003 | ○ | ○ | ○ | ○ | ○ | ○ |
| Estève et al, 1996 | ○ | ○ | ○ | ○ | ○ | ○ |
| Van Doorn et al, 1982 | ○ | ○ | ○ | ○ | ○ | ○ |

| ***Outcome:***  ***HVS symptoms*** | *D1* | *D2* | *D3* | *D4* | *D5* | *Overall* |
| --- | --- | --- | --- | --- | --- | --- |
| *Smyth et al, 2021* | *○* | *○* | *○* | *○* | *○* | *○* |
| *van Gestel et al, 2012* | *○* | *○* | *○* | *○* | *○* | *○* |
| *Collins et al, 2008* | *○* | *○* | *○* | *○* | *○* | *○* |
| *Collins et al, 2003* | *○* | *○* | *○* | *○* | *○* | *○* |
| *Estève et al, 1996* | *○* | *○* | *○* | *○* | *○* | *○* |
| *Van Doorn et al, 1982* |  |  |  |  |  |  |

| **Outcome:**  **Oxygen Consumption** | D1 | D2 | D3 | D4 | D5 | Overall |
| --- | --- | --- | --- | --- | --- | --- |
| Smyth et al, 2021 | ○ | ○ | ○ | ○ | ○ | ○ |
| van Gestel et al, 2012 | ○ | ○ | ○ | ○ | ○ | ○ |
| Collins et al, 2008 |  |  |  |  |  |  |
| Collins et al, 2003 |  |  |  |  |  |  |
| Estève et al, 1996 | ○ | ○ | ○ | ○ | ○ | ○ |
| Van Doorn et al, 1982 | ○ | ○ | ○ | ○ | ○ | ○ |

| **Outcome:**  **Ventilatory parameters** | D1 | D2 | D3 | D4 | D5 | Overall |
| --- | --- | --- | --- | --- | --- | --- |
| Smyth et al, 2021 |  |  |  |  |  |  |
| van Gestel et al, 2012 | ○ | ○ | ○ | ○ | ○ | ○ |
| Collins et al, 2008 |  |  |  |  |  |  |
| Collins et al, 2003 |  |  |  |  |  |  |
| Estève et al, 1996 | ○ | ○ | ○ | ○ | ○ | ○ |
| Van Doorn et al, 1982 | ○ | ○ | ○ | ○ | ○ | ○ |

| **Outcome:**  **Inspiratory capacity** | D1 | D2 | D3 | D4 | D5 | Overall |
| --- | --- | --- | --- | --- | --- | --- |
| Smyth et al, 2021 | ○ | ○ | ○ | ○ | ○ | ○ |
| van Gestel et al, 2012 | ○ | ○ | ○ | ○ | ○ | ○ |
| Collins et al, 2008 |  |  |  |  |  |  |
| Collins et al, 2003 |  |  |  |  |  |  |
| Estève et al, 1996 | ○ | ○ | ○ | ○ | ○ | ○ |
| Van Doorn et al, 1982 | ○ | ○ | ○ | ○ | ○ | ○ |

| **Outcome:**  **FEV_1_ and FVC** | D1 | D2 | D3 | D4 | D5 | Overall |
| --- | --- | --- | --- | --- | --- | --- |
| Smyth et al, 2021 | ○ | ○ | ○ | ○ | ○ | ○ |
| van Gestel et al, 2012 |  |  |  |  |  |  |
| Collins et al, 2008 | ○ | ○ | ○ | ○ | ○ | ○ |
| Collins et al, 2003 | ○ | ○ | ○ | ○ | ○ | ○ |
| Estève et al, 1996 |  |  |  |  |  |  |
| Van Doorn et al, 1982 | ○ | ○ | ○ | ○ | ○ | ○ |

| **Outcome:**  **End Tidal PCO_2_** | D1 | D2 | D3 | D4 | D5 | Overall |
| --- | --- | --- | --- | --- | --- | --- |
| Smyth et al, 2021 | ○ | ○ | ○ | ○ | ○ | ○ |
| van Gestel et al, 2012 | ○ | ○ | ○ | ○ | ○ | ○ |
| Collins et al, 2008 | ○ | ○ | ○ | ○ | ○ | ○ |
| Collins et al, 2003 | ○ | ○ | ○ | ○ | ○ | ○ |
| Estève et al, 1996 | ○ | ○ | ○ | ○ | ○ | ○ |
| Van Doorn et al, 1982 |  |  |  |  |  |  |

| **Outcome:**  **Perceived**  **breathlessness** | D1 | D2 | D3 | D4 | D5 | Overall |
| --- | --- | --- | --- | --- | --- | --- |
| Smyth et al, 2021 | ○ | ○ | ○ | ○ | ○ | ○ |
| van Gestel et al, 2012 |  |  |  |  |  |  |
| Collins et al, 2008 |  |  |  |  |  |  |
| Collins et al, 2003 |  |  |  |  |  |  |
| Estève et al, 1996 | ○ | ○ | ○ | ○ | ○ | ○ |
| Van Doorn et al, 1982 | ○ | ○ | ○ | ○ | ○ | ○ |

| **Outcome:**  **Mastery** | D1 | D2 | D3 | D4 | D5 | Overall |
| --- | --- | --- | --- | --- | --- | --- |
| Smyth et al, 2021 | ○ | ○ | ○ | ○ | ○ | ○ |
| van Gestel et al, 2012 |  |  |  |  |  |  |
| Collins et al, 2008 |  |  |  |  |  |  |
| Collins et al, 2003 | ○ | ○ | ○ | ○ | ○ | ○ |
| Estève et al, 1996 | ○ | ○ | ○ | ○ | ○ | ○ |
| Van Doorn et al, 1982 | ○ | ○ | ○ | ○ | ○ | ○ |

| **Outcome:**  **Emotional function** | D1 | D2 | D3 | D4 | D5 | Overall |
| --- | --- | --- | --- | --- | --- | --- |
| Smyth et al, 2021 | ○ | ○ | ○ | ○ | ○ | ○ |
| van Gestel et al, 2012 |  |  |  |  |  |  |
| Collins et al, 2008 |  |  |  |  |  |  |
| Collins et al, 2003 | ○ | ○ | ○ | ○ | ○ | ○ |
| Estève et al, 1996 | ○ | ○ | ○ | ○ | ○ | ○ |
| Van Doorn et al, 1982 | ○ | ○ | ○ | ○ | ○ | ○ |

| **Outcome:**  **Perceived exertion/**  **fatigue** | D1 | D2 | D3 | D4 | D5 | Overall |
| --- | --- | --- | --- | --- | --- | --- |
| Smyth et al, 2021 | ○ | ○ | ○ | ○ | ○ | ○ |
| van Gestel et al, 2012 |  |  |  |  |  |  |
| Collins et al, 2008 |  |  |  |  |  |  |
| Collins et al, 2003 |  |  |  |  |  |  |
| Estève et al, 1996 | ○ | ○ | ○ | ○ | ○ | ○ |
| Van Doorn et al, 1982 | ○ | ○ | ○ | ○ | ○ | ○ |

| **Outcome:**  **Exercise duration/capacity** | D1 | D2 | D3 | D4 | D5 | Overall |
| --- | --- | --- | --- | --- | --- | --- |
| Smyth et al, 2021 | ○ | ○ | ○ | ○ | ○ | ○ |
| van Gestel et al, 2012 |  |  |  |  |  |  |
| Collins et al, 2008 |  |  |  |  |  |  |
| Collins et al, 2003 |  |  |  |  |  |  |
| Estève et al, 1996 | ○ | ○ | ○ | ○ | ○ | ○ |
| Van Doorn et al, 1982 | ○ | ○ | ○ | ○ | ○ | ○ |

ROBINS-I: Risk of Bias summaries by outcome:

ROBINS I considers the risk of bias in non-randomised studies looking at the effects of interventions using seven domains to identify an overall risk of bias judgement. Risk is defined as low risk of bias, moderate risk of bias, serious risk of bias, critical risk of bias and no information

**No information**	**Moderate**	**Serious	○ Outcome not measured**

**Domains: D1: Bias due to confounding; D2: Bias due to selection of participants; D3: Bias in classification of interventions; D4: Bias due to deviations from intended interventions; D5: Bias due to missing data; D6: Bias in measurement of outcomes; D7: Bias in selection of the reported result.**

| **Outcome:**  **TAM/CWS** | D1 | D2 | D3 | D4 | D5 | D6 | D7 | Overall |
| --- | --- | --- | --- | --- | --- | --- | --- | --- |
| Ishii et al, 2021 |  |  |  |  |  |  |  |  |
| Sackner et al, 1984 |  |  |  |  |  |  |  |  |

| **Outcome:**  **Oxygen consumption** | D1 | D2 | D3 | D4 | D5 | D6 | D7 | Overall |
| --- | --- | --- | --- | --- | --- | --- | --- | --- |
| Ishii et al, 2021 |  |  |  |  |  |  |  |  |
| Sackner et al, 1984 | ○ | ○ | ○ | ○ | ○ | ○ | ○ | ○ |

| **Outcome:**  **Ventilatory parameters** | D1 | D2 | D3 | D4 | D5 | D6 | D7 | Overall |
| --- | --- | --- | --- | --- | --- | --- | --- | --- |
| Ishii et al, 2021 |  |  |  |  |  |  |  |  |
| Sackner et al, 1984 |  |  |  |  |  |  |  |  |
