# Supplementary material for: Biofeedback in Breathing Pattern Training: A Systematic Review
Source: Can Respir J. 2026 Nov 15;2026:5567144. doi: 10.1155/carj/5567144 (PMC13770288; doi:10.1155/carj/5567144)
Supplement: Supplementary file 6 [file CARJ-2026-5567144-s006.docx]

**Supplementary Material 7. Risk of bias and GRADE Decision Rationale	RoB-2: Summaries**

| **Domain** | **Assessments by**  **outcome** | **Comment** |
| --- | --- | --- |
| **Risk of bias arising from the**  **randomization process** | 0_1_ Some concerns | Study protocol just states that participants were randomly assigned to the experimental or control group. Discussion with co-author identified that a computer-generated random number package was used. No significant differences in baseline measurements between groups. Process of randomisation sound. Intervention allocation not externally or independently sourced (concealed) but allocation randomly assigned by computer generator, therefore selection bias reduced. Therefore, decision made to stratify as some concerns rather than high risk. |
|  | 0_2_ Some concerns | As for comment above. |
| **Risk of bias due to deviations**  **from the intended interventions** | 0_1_ Some concerns | Likely that participants, people delivering the intervention and carers aware of the assigned intervention. No deviation to planned interventions identified. Although researchers were aware of the intervention group all controllable factors consistent across groups in this acute interventional study. |
|  | 0_2_ Some concerns | As for comment above. |
| **Missing outcome data** | 0_1_ Low | Data for this outcome was complete and reported. |
|  | 0_2_ Low | As for comment above. |
| **Risk of bias in measurement**  **of the outcome** | 0_1_ Low | It is appropriate to measure ventilatory parameters when investigating interventions for dysfunctional breathing patterns. Outcome measurements and data collection appear consistent between groups. Physiological measurements in this study likely not to be influenced by knowledge of intervention received. |
|  | 0_2_ Low | Optoelectronic plethysmography widely used to measure chest wall movement and synchrony and is reported in numerous studies. Measurement of outcomes consistent between groups and appropriate for intervention. Physiological measurements in this study likely not to be influenced by knowledge of intervention received. |
| **Risk of bias in selection of the**  **reported result** | 0_1_ Low | Ethics application sited and consistent with publication. Results correspond to the intended outcome measures. No option for the opportunity to select alternative or multiple measures. Outcomes measured pre and post intervention in intended way. Outcomes not analysed in multiple ways. A priori plan sited. Results correspond to outcome measures and planned analysis therefore stratified as low risk. |
|  | 0_2_ Low | As for comment above |
| **Overall risk of bias** | 0_1_ Some concerns | Stratified as some concerns in at least one domain, therefore overall risk of bias for this study is some concerns. |
|  | 0_2_ Some concerns |  |

***Smyth et al, (2021): (O_1_ = ventilatory parameters; O_2_ = thoracoabdominal movement/ chest wall synchrony)***

***Van Gestel et al, (2012): (O_1_ = exercise duration; O_2_ = QOL- breathlessness; O_3_ = QOL- fatigue; O_4_ = QOL- emotion; O_5_ = QOL- mastery; O_6_ = forced expiratory volume in 1 minute/ forced vital capacity (FEV_1_/FVC)): QOL= Quality of Life***

| **Domain** | **Assessments by**  **outcome** | **Comment** |
| --- | --- | --- |
| **Risk of bias arising from the**  **randomization process** | 0_1_ Some concerns | Study protocol just states that it is a randomised controlled trial. No details presented on randomization method or regarding allocation concealment. No significant between group differences noted. |
|  | 0_2_ Some concerns | As for comment above. |
|  | O_3_ Some concerns | As for comment above. |
|  | O_4_ Some concerns | As for comment above. |
|  | O_5_ Some concerns | As for comment above. |
|  | O_6_ Some concerns | As for comment above. |
| **Risk of bias due to deviations**  **from the intended interventions** | 0_1_ High | Participants in this study took part in conventional pulmonary rehab or conventional pulmonary rehab plus controlled breathing (RBF). Therefore participants, carers and people delivering the intervention were likely aware of the assigned intervention. There is no information presented regarding non-protocol interventions. For example, how much exercise participants took part in outside of the study or other factors which could affect the outcome. No information is presented regarding adherence or completion. Study limitation highlighted participant difficulty in changing breathing pattern during exercise. No information on effect estimate analysis presented. |
|  | 0_2_ High | As for comment above. |
|  | O_3_ High | As for comment above. |
|  | O_4_ High | As for comment above. |
|  | O_5_ High | As for comment above. |
|  | O_6_ High | As for comment above. |
| **Missing outcome data** | 0_1_ Low | Data for this outcome was complete and reported. Data for all participants presented as group means. No obvious omissions detected. |
|  | 0_2_ Low | As for comment above. |
|  | O_3_ Low | As for comment above. |
|  | O_4_ Low | As for comment above. |
|  | O_5_ Low | As for comment above. |
|  | O_6_ Low | As for comment above. |
| **Risk of bias in measurement**  **of the outcome** | 0_1_ Low | It is appropriate to measure exercise duration when investigating the effects of respiratory biofeedback. Outcome measurements and data collection appear consistent between groups. 6-minute walking test (6MWT) is an appropriate measure of exercise capacity and a validated measure. There is standard guidance on test implementation. Technicians performed the 6MWT and were blinded to randomization. |
|  | 0_2_ Low | Quality of life measurements are appropriate to measure when investigating respiratory biofeedback. Patient reported outcome. Standardised instructions for completion. No information as to if assessors were blinded but the standardisation of this test would reduce influence. The subjective nature of this measurement is noted. |
|  | O_3_ Low | As for comment above. |
|  | O_4_ Low | As for comment above. |
|  | O_5_ Low | As for comment above. |
|  | O_6_ Low | It is appropriate to measure pulmonary function when investigation respiratory biofeedback. Comments as for O_1_. |
| **Risk of bias in selection of the**  **reported result** | 0_1_ Some concerns | Study registered with ClinicalTrials.gov identifier: NCT01175265. Study start date reported to be Nov 1, 2008, and completion date reported to be April 1, 2010. The first registration of this study with ClinicalTrials.gov was July 13, 2010. Therefore, this suggests that the study was completed before registration and an a priori plan cannot therefore be guaranteed. Multiple outcome measurements and analyses likely not used. |
|  | 0_2_ Some concerns | As for comment above |
|  | O_3_ Some concerns | As for comment above |
|  | O_4_ Some concerns | As for comment above |
|  | O_5_ Some concerns | As for comment above |
|  | O_6_ Some concerns | As for comment above |
| **Overall risk of bias** | 0_1_ High | Stratified as high in one domain, therefore overall risk of bias for this study is high. |
|  | 0_2_ High |  |
|  | O_3_ High |  |
|  | O_4_ High |  |
|  | O_5_ High |  |
|  | O_6_ High |  |

***Collins et al, (2008): (O_1_ = exercise duration; O_2_ = ventilatory parameters; O_3_ = inspiratory capacity; O_4_ = oxygen consumption; O_5_ = quality of life (QOL)- breathlessness; O_6_ = QOL- fatigue; O_7_ = QOL- emotion; O_8_ = QOL- mastery)***

| **Domain** | **Assessments by**  **outcome** | **Comment (on-line supplements also consulted)** |
| --- | --- | --- |
| **Risk of bias arising from the**  **randomization process** | O_1_ Some concerns | Method of randomisation outlined (permuted block). Information on allocation procedure not available. Randomisation judged to be sound. No information on allocation sequence. Baseline differences not significant therefore judged to be some concerns. |
|  | O_2_ Some concerns | As for comment above |
|  | O_3_ Some concerns | As for comment above |
|  | O_4_ Some concerns | As for comment above |
|  | O_5_ Some concerns | As for comment above |
|  | O_6_ Some concerns | As for comment above |
|  | O_7_ Some concerns | As for comment above |
|  | O_8_ Some concerns | As for comment above |
| **Risk of bias due to deviations**  **from the intended interventions** | O_1_ Some concerns | It was likely that participants were aware of their assigned intervention, but it is not known if they were aware of the other available interventions. Researchers delivering intervention were likely aware of what protocol was being delivered. Other factors which may affect measured outcomes in a population with chronic obstructive pulmonary disease are participant activity levels outside of study protocol, changes to medication, attendance to pulmonary rehabilitation. No information is available on this. Interventions implemented as planned. 15 participants dropped out of this study. Rationale for drop out given. Demographic details of drop out compared and no significant difference identified. ANCOVA (analysis of variance) and intention to treat analysis outlined. |
|  | O_2_ Some concerns | As for comment above |
|  | O_3_ Some concerns | As for comment above |
|  | O_4_ Some concerns | As for comment above |
|  | O_5_Some concerns | As for comment above |
|  | O_6_ Some concerns | As for comment above |
|  | O_7_ Some concerns | As for comment above |
|  | O_8_ Some concerns | As for comment above |
| **Missing outcome data** | O_1_ Some concerns | 15 participants dropped out (withdrew or withdrawn) out after randomisation (23%). |
|  | O_2_ Some concerns | As for comment above |
|  | O_3_ Some concerns | Inspiratory capacity (IC) data lost in the first cohort of the study (n=13) but due to a technical issue and explained. Data were lost for all groups and not just one. IC analysis based on 12 participants in each group. Judged likely to not bias results based on consensus. |
|  | O_4_ Some concerns | As for comment above |
|  | O_5_ Some concerns | As for comment above |
|  | O_6_ Some concerns | As for comment above |
|  | O_7_ Some concerns | As for comment above |
|  | O_8_ Some concerns | As for comment above |
| **Risk of bias in measurement**  **of the outcome** | O_1_ Low | Exercise duration is an appropriate measure when looking at ventilation feedback training. Outcome measurements and data collection appear consistent between groups. Likely that researchers were aware of the interventions received by participants. Likely that physiological measurements could not be influenced. On-line supplement outlines standard encouragement for testing. |
|  | O_2_ Low | Ventilatory parameters (respiratory pattern) is an appropriate measure when looking at ventilation feedback training. As for comment above |
|  | O_3_ Low | Exercise induced dynamic hyperinflation is an appropriate measure when looking at ventilation feedback training. As for comment above. |
|  | O_4_ Low | Oxygen consumption is an appropriate measure when looking at ventilation feedback. As for comment above. |
|  | O_5_ Low | Perceived breathlessness is appropriate to measure when investigating ventilation biofeedback. QOL measures are patient reported outcomes. Standardised instructions for completion. No information as to if assessors were blinded but the standardisation of this test would reduce influence. The subjective nature of this measurement is noted. |
|  | O_6_ Low | Fatigue measures are an appropriate measure when looking at ventilation feedback. As for comments O_5_. |
|  | O_7_ Low | Emotion is appropriate to measure when investigating ventilation feedback. As for comments O_5_. |
|  | O_8_ Low | Mastery is appropriate to measure when investigation ventilation feedback. As for comments O_5_. |
| **Risk of bias in selection of**  **the reported result** | O_1_ Low | From review of on-line supplement data presumed to be analysed in accordance with a pre-specified plan. Outcomes measured in one way at determined timepoints. Outcomes measured pre and post intervention in intended way. Outcomes not analysed in multiple ways. |
|  | O_2_ Low | As for comment above |
|  | O_3_ Low | As for comment above |
|  | O_4_ Low | As for comment above |
|  | O_5_ Low | As for comment above |
|  | O_6_ Low | As for comment above |
|  | O_7_ Low | As for comment above |
|  | O_8_ Low | As for comment above |
| **Overall risk of bias** | O_1_ Some concerns | Overall risk of bias is High . |
|  | O_2_ Some concerns |  |
|  | O_3_ Some concerns |  |
|  | O_4_ Some concerns |  |
|  | O_5_ Some concerns |  |
|  | O_6_ Some concerns |  |
|  | O_7_ Some concerns |  |
|  | O_8_ Some concerns |  |

***Collins et al, (2003): (O_1_ = exercise duration; O_2_ = perceived breathlessness; O_3_ = perceived exertion; O_4_ = ventilatory parameters; O_5_ = oxygen consumption; O_6_ = inspiratory capacity)***

| **Domain** | **Assessments by**  **outcome** | **Comment** |
| --- | --- | --- |
| **Risk of bias arising from the**  **randomization process** | O_1_ Some concerns | Method states participants randomly assigned to groups, but no other explanation detailed. However, this comes from an established research group so a decision for PY made. Smoking pack year history lower in the ventilation feedback with exercise group but reported not to be significantly different. Randomisation judged to be sound. No information on allocation sequence. Baseline differences not significant therefore judged to be some concerns. |
|  | O_2_ Some concerns | As for comment above |
|  | O_3_ Some concerns | As for comment above |
|  | O_4_ Some concerns | As for comment above |
|  | O_5_ Some concerns | As for comment above |
|  | O_6_ Some concerns | As for comment above |
| **Risk of bias due to deviations**  **from the intended interventions** | O_1_ Some concerns | It was likely that participants were aware of their assigned intervention, but it is not known if they were aware of the other available interventions. Researchers delivering intervention were aware of what protocol was being delivered. No information was provided as to whether participants attended pulmonary rehabilitation as well, or what their activity levels were during the 6- week study other than the prescribed exercise. No information as to whether there was any change to regular medications. Participants needed to be infection free for 4 weeks, but it did not mention if they had to be exacerbation free (non-infective). These could affect outcome. Interventions implemented as planned. 8 participants dropped out of this study (2 after randomisation). Rationale for drop out given. Demographic details of drop out compared and no significant difference identified, though method of analysis not described. |
|  | O_2_ Some concerns | As for comment above |
|  | O_3_ Some concerns | As for comment above |
|  | O_4_ Some concerns | As for comment above |
|  | O_5_ Some concerns | As for comment above |
|  | O_6_ Some concerns | As for comment above |
| **Missing outcome data** | O_1_ Some concerns | Data for this outcome available for all participants that completed the study. However, 8 subjects dropped out (two who were randomised). The two participants that left after randomisation left due to reasons unrelated to the study. No significant difference in demographic data between those that dropped out and those who completed. |
|  | O_2_ Some concerns | As for comment above |
|  | O_3_ Some concerns | As for comment above |
|  | O_4_ Some concerns | As for comment above |
|  | O_5_ Some concerns | As for comment above |
|  | O_6_ Some concerns | As for comment above |
| **Risk of bias in measurement**  **of the outcome** | O_1_ Low | Exercise endurance is an appropriate measure when looking at biofeedback and breathing. Outcome measurements and data collection appear consistent between groups. Researchers were aware of the interventions received by participants. Likely that physiological measurements could not be influenced. Standardise instruction given for exercise tests. Measurement of outcomes presumed to be consistent between groups and appropriate for intervention. |
|  | O_2_ Some concerns | Perceived breathlessness is appropriate to measure when investigating ventilation biofeedback. QOL measures are patient reported outcomes. No information as to if assessors were blinded but BORG scale open to influence. The subjective nature of this measurement is noted. |
|  | O_3_ Some concerns | As for O_2_. |
|  | O_4_Low | Ventilatory parameters are an appropriate measure when looking at biofeedback and breathing. As for O_1_above. |
|  | O_5_ Low | Oxygen consumption is an appropriate measure when looking at biofeedback and breathing. As for O_1_ above. |
|  | O_6_ Low | Inspiratory capacity is an appropriate measure to consider when looking at biofeedback and breathing. As for O_1_above. |
| **Risk of bias in selection of**  **the reported result** | O_1_ Some concerns | No pre-specified analysis plan sited or referenced. Outcomes measured pre and post intervention in intended way. Outcomes not analysed in multiple ways. |
|  | O_2_ Some concerns | As for comment above |
|  | O_3_ Some concerns | As for comment above |
|  | O_4_ Some concerns | As for comment above |
|  | O_5_ Some concerns | As for comment above |
|  | O_6_ Some concerns | AS for comment above |
| **Overall risk of bias** | O_1_ Some concerns | Overall risk of bias is some concerns as stratified this in more than one domain but judged not to be critical. |
|  | O_2_ Some concerns |  |
|  | O_3_ Some concerns |  |
|  | O_4_ Some concerns |  |
|  | O_5_ Some concerns |  |
|  | O_6_ Some concerns |  |

***Estève et al, (1996): (O_1_ = FEV_1_ and FVC)***

| **Domain** | **Assessments by**  **outcome** | **Comment** |
| --- | --- | --- |
| Risk of bias arising from the  randomization process | O_1_ High | Study protocol says that patients were selected from the pulmonary rehabilitation centre according to criterion, but it does not describe how. Study protocol just states that participants were randomly assigned to the experimental or control group. Intervention allocation not externally or independently sourced. Inadequate information on allocation procedure. Breathing pattern training (BPT) group were younger (Mean age 66) and (77) in control group. As inadequate information regarding randomisation presents a decision of PY made for 1.3. Process of randomisation unclear. Not enough information is available to inform decision making. Researcher judgement gives a potential for high risk, therefore stratified as so. |
| Risk of bias due to deviations  from the intended interventions | O_1_ High | This study either had an intervention for BPT or no intervention (usual care). It is likely that researchers delivering intervention and participants were aware of what protocol was being delivered. It was unable to be determined whether important non-protocol interventions were balanced across groups as all participants were inpatients undergoing daily physiotherapy, bronchodilators and pulmonary rehab but the extend of this is unknown. Algorithm stratification high. |
| Missing outcome data | O_1_ High | Data were accidentally lost for one experimental group patient which is 10% of that group. No further details of the data lost described. Overall, data is 95% complete but low study numbers and no further explanation or reference to data loss present. There is not enough information to stratify other than high in this domain. |
| Risk of bias in measurement  of the outcome | O_1_ High | It is appropriate to measure forced expiratory volume in 1 second (FEV_1_) and forced vital capacity (FVC) when investigating dysfunctional breathing pattern training. This is best measured by spirometry. Outcome measurements and data collection appear consistent between groups. It is likely that researchers were aware of the interventions received by participants. It is unclear who performed the spirometry in this study which could influence outcome. Algorithm stratification high. |
| Risk of bias in selection of  the reported result | O_1_ Some concerns | No pre-specified analysis plan available or referenced. Outcomes measured at two timepoints. Both reported. Outcomes measured pre and post intervention in intended way. Outcomes not analysed in multiple ways. Note respiratory inductance plethysmography data not reported in the study. |
| **Overall risk of bias** | O_1_ High | Judged to be at high risk of bias in more than one domain. Of note: patients were in-patients in a rehabilitation centre and were also receiving daily chest physio and bronchodilators. Therefore, this may also have impacted lung function and the primary outcomes of this study. Therefore, difficult to know if BPT led to improvement in FEV_1_ and FVC. No results for respiratory inductance plethysmography published which were also measured. |

***Van Doorn et al, (1982): (O_1_ = end tidal partial pressure of carbon dioxide (PCO_2_); O_2_ = hyperventilation syndrome symptoms)***

| **Domain** | **Assessments by**  **outcome** | **Comment** |
| --- | --- | --- |
| **Risk of bias arising from the**  **randomization process** | 0_1_ High | Method states participants randomly assigned to groups, but no other explanation detailed. Intervention allocation not externally or independently sourced. Baseline differences between groups (end-tidal PCO_2_ and symptoms) evident. Biofeedback group had higher PCO_2_ and symptom scores at baseline. Authors wrote “Did not succeed completely in matching the two groups on end tidal PCO_2_” Therefore, stratified as high risk of bias in this domain. |
|  | 0_2_ High | As above |
| **Risk of bias due to deviations**  **from the intended interventions** | 0_1_ High | It is likely that participants were aware of their assigned intervention as biofeedback was being compared to breathing instruction. Experimental group had therapist led sessions. Breathing training group had some independent home-based training. Likely that researchers delivering intervention were aware of what protocol was being delivered, though interventions implemented as planned. |
|  | 0_2_ High | As above |
| **Missing outcome data** | 0_1_ Low | Data for this outcome available for all participants. No missing outcome data |
|  | 0_2_ Low | As above |
| **Risk of bias in measurement**  **of the outcome** | 0_1_ High | End tidal partial pressure of carbon dioxide (PCO_2_) is an appropriate measure when looking at biofeedback and breathing. Outcome measurements and data collection appear consistent between groups. Likely that researchers were aware of the interventions received by participants. PCO_2_ can be affected by RR, stress, anxiety, breath holding etc. Unable to determine if standardisation present during measurement, therefore NI for 4.4 and 4.5. |
|  | 0_2_ Some concerns | A symptoms score would be appropriate to investigate for this intervention. Measurement of outcomes presumed to be consistent between groups and appropriate for intervention. Researchers were aware of the interventions received by participants. This is a patient reported outcome and element of judgment present. Symptom data collected by a team from a separate discipline to reduce bias. |
| **Risk of bias in selection of the**  **reported result** | 0_1_ Some concerns | Unable to determine if trial analysed in accordance with a pre-specified plan. Outcomes measured pre and post intervention in intended way. Outcomes not analysed in multiple ways. |
|  | 0_2_ Some concerns | As above |
| **Overall risk of bias** | 0_1_ High | Stratified as high risk in more than one domain. |
|  | 0_2_ High |  |

**Risk of bias profiles ROBINS-I : Summaries**

**Ishii et al, (2021) (O_1_ = oxygen consumption (VO_2_/BW); O_2_ = thoracoabdominal movement; O_3_ = ventilatory parameters)**

| There does not appear to be a pre-intervention prognostic factor that predicts whether an individual receives one or the other intervention. No relevant co-interventions identified by systematic review research team which may affect outcome for this study in this setting. | | |
| --- | --- | --- |
| **Domain** | **Assessments by**  **outcome** | **Comment** |
| Bias due to confounding | O_1_ Moderate | This describes a pilot study with an acute intervention with healthy subjects. Baseline confounding not identified to be significant. Though, participant characteristics such as physical fitness and baseline measures might influence results. Other factors that could have a potential effect on outcome measures such as acute activity and stress likely to be addressed from study protocol. Room characteristics (temp/humidity) etc likely to be constant. Serious residual confounding not suspected therefore scored moderate risk. On balance, with this set of healthy participants, in this setting, confounding considered to be moderate. |
|  | O_2_ Moderate | As above |
|  | O_3_ Moderate | As above |
| Bias in selection of participants  into the study | O_1_ No information | Selection into the study not determined by subject characteristics observed after the start of the study. No information on recruitment or inclusion/ exclusion. All participants are male and young adults and appear to be volunteers. No information on level of fitness, minimal information on past medical history, no information on current medications which could all influence this outcome. |
|  | O_2_ No information | As above |
|  | O_3_ No information | As above |
| Bias in classification of  interventions | O_1_Moderate | Pilot study. Experimental protocols and interventions clear. Information collected at the time of intervention. |
|  | O_2_ Moderate | As above |
|  | O_3_ Moderate | As above |
| Bias due to deviations from  intended interventions | O_1_ Moderate | No deviations suggested in the study. One intervention group, therefore, no systematic differences in care provided between groups beyond assigned interventions. Therefore, scored as moderate risk in this domain. |
|  | O_2_ Moderate | As above |
|  | O_3_ Moderate | As above |
| Bias due to missing data | O_1_ Moderate | Data set complete but small number of participants. |
|  | O_2_ Moderate | As above |
|  | O_3_ Moderate | As above |
| Bias in measurement of  outcomes | O_1_ Moderate | Assessors aware of the intervention. Outcome assessments comparable across participants. VO_2_/BW measures physiological parameters and would not be significantly influenced by knowledge of intervention received, therefore scored as moderate risk in this domain. |
|  | O_2_ Moderate | Thoracoabdominal movement could be influenced by participant knowledge, though on balance deemed not to be significant. |
|  | O_3_ Moderate | As above |
| Bias in selection of the  reported result | O_1_ Moderate | No a priori protocol. Analyses and outcome seem appropriate for this study within the setting. No evidence of multiple selection of measurement or analyses. The absence of a pre-registered analysis plan does cause some doubt |
|  | O_2_ Moderate | As above |
|  | O_3_ Moderate | As above |
| **Overall risk of bias** | O_1_ No information | Stratified as no information as judged to be a lack of information in one domain. |
|  | O_2_ No information |  |
|  | O_3_ No information |  |

**Sackner et al, (1984) (O_1_ = ventilatory parameters; O_2_ = thoracoabdominal movement/ chest wall synchrony)**

| This study uses subjects as their own control (pre and post intervention). Although, both intervention groups received biofeedback, there was some protocol difference depending on diagnosis evident at pre-intervention. The group considered as normal additionally had time for thoracic breathing and thoracic breathing with feedback. Therefore, chronic obstructive pulmonary disease (COPD) appears to be a baseline confounder in this study as it determined study protocol. No attempt made to match subjects for age, diagnosis or lung function. No relevant co-interventions identified which may affect outcome in this setting, for this study. | | |
| --- | --- | --- |
| **Domain** | **Assessments by**  **outcome** | **Comment** |
| Bias due to confounding | O_1_ Serious | Baseline confounding not controlled for in this study. Serious residual confounding not anticipated. Overall, it is unclear if all important confounding domains were likely to be addressed in this study therefore stratified as serious risk of bias. |
|  | O_2_ Serious | As above |
| Bias in selection of participants  into the study | O_1_ No information | Minimal information is reported about selection of participants into the study. Normal groups not defined at all and patients with COPD defined as eucapnic or slightly hypocapnic. Minimal anthropometric and spirometry data given. Selection and recruitment is unclear and on balance it was felt that there was not enough information to base a judgement on this domain. Outcomes could be affected by past medical history, fitness and previous exposure. |
|  | O_2_ No information | As above |
| Bias in classification of  interventions | O_1_ Moderate | Interventions clearly outlined |
|  | O_2_ Moderate | As above |
| Bias due to deviations from  intended interventions | O_1_ Moderate | Presumed to be no deviations in the study. No systematic differences in care provided between groups beyond assigned interventions suggested, therefore scored as moderate risk in this domain. |
|  | O_2_ Moderate | As above |
| Bias due to missing data | O_1_ No information | Small data set. 10% less participants in COPD group for which is not accounted. Some potential for data to be missing but not enough information to answer fully. |
|  | O_2_ No information | As above |
| Bias in measurement of  outcomes | O_1_ Moderate | Assessors aware of the intervention. Outcome assessments comparable across groups. Breath by breath analysis measures physiological parameters and would not be significantly influenced by knowledge of intervention received, therefore scored as moderate risk in this domain. |
|  | O_2_ Moderate | Respiratory inductance plethysmography measures physiological parameters and on balance would not be significantly influenced by knowledge of intervention received. |
| Bias in selection of the  reported result | O_1_ Moderate | No a priori protocol. Analyses and outcome seem appropriate for this study within the setting. Likely that multiple outcomes and analyses were not used but not 100% assured as no pre-specified analysis plan or trial registry. |
|  | O_2_ Moderate | As above |
| **Overall risk of bias** | O_1_ Serious | Stratified as at serious risk of bias as judged to score this in one domain and a lack of information in two domains. |
|  | O_2_ Serious |  |

**GRADE Evidence Profile Table-Summary** This system considers five key areas to make a judgement about quality (risk of bias, inconsistency, indirectness, imprecision, and publication bias). Ratings range from high to very low and usually start with a baseline rating of high for randomised controlled trials (RCT’s) and low for non-randomised controlled trials (NRCT's).

| **Outcome**  **Number of participants and (studies)** | **Study** | **Number of participants** | **Study design** | **Risk of bias**  **(ROB-2 or ROBINS-I)** | **Inconsistency** | **Indirectness** | **Imprecision** | **Publication bias** | **Quality of evidence for OUTCOME and justifications for downgrading** |
| --- | --- | --- | --- | --- | --- | --- | --- | --- | --- |
| Thoraco-abdominal movement (TAM)/ chest wall synchrony (CWS)  45 (3) | Smyth *et al,* 2021 | 18 | Randomised controlled trial (RCT) | Some concerns | There is high clinical and methodological heterogeneity among these three studies  Differences are associated with study design, participants and protocols  Reported results are conflicting | Directly applicable but population specific: subjects with presumed dysfunctional breathing  Visual feedback used | Imprecision significant due to low sample sizes across the studies. | Pre-specified plan available on request via institutional review board  No obvious funding issues or conflict of interest  Innovative studies may have higher chance of publication bias | 3 studies: 1 RCT (high), 1 non-randomised pilot study (low) and one observational study (low)  If outcome based on start high for the RCT, then a downgrade of 3 is made. If outcome based on start low for the non-RCT’s then a downgrade of 1 is made  Downgrade by one level when risk of bias is considered. Downgrade by one level when inconsistency is considered. Evidence is applicable to the review question therefore, no downgrade for indirectness. Imprecision significant, therefore downgrade by one level. Publication bias also a factor. One innovative study, one pilot study and one published in 1984  Reasons to upgrade not evident  **Rating: Very low** |
|  | Ishii *et al,* 2021 | 8 | Non-randomised pilot study | No information |  | Directly applicable but population specific: healthy subjects  Visual feedback used |  | No a priori analysis plan available  No obvious funding issues or conflict of interest. Research supported by Japan Society for the Promotion of Science  Pilot studies may have higher chance of publication bias |  |
|  | Sackner *et al,* 1984 | 19 | Comparative observational study | Serious |  | Directly applicable but population specific: subjects with COPD  Visual feedback used |  | No a priori analysis plan available.  No obvious funding issues or conflict of interest. Research supported by the National Heart, Lung and Blood Institute and the Jefferson L Ford III memorial foundation |  |
| HVS symptoms  1 (20) | Van Doorn *et al,* 1982 | 20 | RCT | High | Not applicable-single study | Directly applicable but population specific: subjects with hyperventilation syndrome (HVS)  Visual feedback used | Imprecision significant due to low sample size | No a priori plan available  Study published in 1982  No obvious funding issues or conflict of interest. Research supported by the Netherlands Asthma Foundation | Start high (RCT)  One study for this outcome. Rated high risk of bias, downgrade by one level. Evidence is appropriate for review question, therefore no downgrade in indirectness. Small sample size and high imprecision, therefore, downgrade one level. No priori plan available and published in 1982. Downgrade 1 level for potential publication bias  Reason to upgrade not evident  **Rating: very low** |
| Oxygen consumption  96 (3) | Collins *et al,* 2008 | 49 | RCT | Some concerns | No serious inconsistency between the two RCT’s  Increased clinical and methodological inconsistency when the non-randomised pilot study considered | Directly applicable but population specific: subjects with moderate to severe COPD  Visual feedback used | Imprecision moderate to significant due to moderate to low sample sizes | Detailed on-line supplement available and pre study registration with [www.clinicaltrials.gov](http://www.clinicaltrials.gov) (NCT 00037973)  No obvious funding issues or conflict of interest. Research supported by the Department of Veteran Affairs, Rehabilitation Research and Development, Advanced Career Development Award | 3 studies, 2 RCT and 1 non-randomised pilot study  Start high: Risk of bias rated as some concerns for 2 studies and no information for 1 study. Therefore, downgraded one level. High inconsistency between the two research groups and limited generalizability of one study. Results conflict, therefore downgraded one level. Moderate to significant imprecision due to sample sizes and results inconsistently reported in one study, therefore downgraded one level. Publication bias could also be a factor as no priori plans available for one RCT and one pilot study  Reason to upgrade not evident  **Rating: very low** |
|  | Collins *et al,* 2003 | 39 | RCT | Some concerns |  | Directly applicable but population specific: subjects with moderate to severe COPD  Visual feedback used |  | No a priori plan available  No obvious funding issues or conflict of interest. Research supported by the Department of Veterans Affairs, Veterans Health Administration, Rehabilitation Research and Development Service |  |
|  | Ishii *et al,* 2021 | 8 | Non-randomised pilot study | No information |  | Limited generalizability in this cohort of healthy non-randomised subjects  Visual feedback used |  | No a priori plan available  No obvious funding issues or conflict of interest. Research supported by Japan Society for the Promotion of Science  Pilot studies may have higher chance of publication bias |  |
| Ventilatory parameters  **Overall**  133 (5)  Tidal volume (VT): 115 (4)  VE:115 (4)  Respiratory rate (RR): 133 (5)  Inspiratory time (Ti): 107 (3)  Expiratory time (Te): 88 (2)  Fractional inspiratory time (Ti/Ttot): 58 (2)  Mean inspiratory flow (Vt/Ti): 19 (1)  Mean expiratory flow (VTE/Te) :19 (1) | Smyth *et al,* 2021  RR | 18 | RCT | Some concerns | There is high clinical and methodological heterogeneity among these five studies  Differences are associated with study design, participants and protocols | Directly applicable but population specific: subjects with presumed dysfunctional breathing  Visual feedback used | Imprecision moderate to significant due to low to moderate sample size. | Pre-specified plan available on request via institutional review board  No obvious funding issues or conflict of interest  Innovative studies may have higher chance of publication bias | 5 studies, 3 RCT, one non-randomised pilot study and one observation study  Start high. Risk of bias with some concerns in 3 studies, no information in one and high in one. Downgrade one level. Significant clinical and methodological heterogeneity in these studies with conflicting results reported (RR, VE, Ti) and single study reporting in (Vt/Ti and VTE/Te). Downgrade one level for inconsistency. Evidence applicable to the review question. Therefore, no downgrade in indirectness indicated. Imprecision moderate to significant due to small/moderate sample sizes, downgrade one level. Mixture of innovate and pilot studies, old data and some without a priori plans. Downgrade one level for publication bias  No reason to upgrade evident.  **Rating Very low**  ***GRADE rating remains very low when outcomes (RR; VT; VE; Ti; Te; Vt/Ti; Ti/Ttot; VTE/Te) are assessed individually, therefore reported together for conciseness** |
|  | Collins et al, 2008  VT  VE  Ti  Te  RR | 49 | RCT | Some concerns |  | Directly applicable but population specific: subjects with moderate to severe COPD  Visual feedback used |  | Detailed on-line supplement available and pre study registration with [www.clinicaltrials.gov](http://www.clinicaltrials.gov) (NCT 00037973)  No obvious funding issues or conflict of interest. Research supported by the Department of Veteran Affairs, Rehabilitation Research and Development, Advanced Career Development Award |  |
|  | Collins *et al,* 2003  VT  VE  Ti  Te  RR  Ti/Ttot | 39 | RCT | Some concerns |  | Directly applicable but population specific: subjects with moderate to severe COPD  Visual feedback used |  | No a priori plan available  No obvious funding issues or conflict of interest. Research supported by the Department of Veterans Affairs, Veterans Health Administration, Rehabilitation Research and Development Service |  |
|  | Ishii *et al,* 2021  VT  VE  RR | 8 | Non-randomised pilot study | No information |  | Limited generalizability in this cohort of healthy non-randomised subjects  Visual feedback used |  | No a priori analysis plan available  No obvious funding issues or conflict of interest. Research supported by Japan Society for the Promotion of Science  Pilot studies may have higher chance of publication bias |  |
|  | Sackner *et al,* 1984  Vt  VE  Ti  RR  Vt/Ti  Ti/Ttot  VTE/Te | 19 | Comparative observational study | Serious concerns |  | Directly applicable but population specific: subjects with COPD  Visual feedback used |  | No a priori analysis plan available  No obvious funding issues or conflict of interest. Research supported by the National Heart, Lung and Blood Institute and the Jefferson L Ford III memorial foundation |  |
| Inspiratory Capacity (IC)  2 (88) | Collins *et al,* 2008 | 49 | RCT | Some concerns | No serious inconsistency between the two RCT’s | Directly applicable but population specific: subjects with moderate to severe COPD  Visual feedback used | Some imprecision due to moderate sample sizes. | Detailed on-line supplement available and pre study registration with [www.clinicaltrials.gov](http://www.clinicaltrials.gov) (NCT 00037973)  No obvious funding issues or conflict of interest. Research supported by the Department of Veteran Affairs, Rehabilitation Research and Development, Advanced Career Development Award | 2 RCT’s start high:  Some concerns for risk of bias, downgrade one level. No serious inconsistency between studies, no downgrade. Directly relevant to research question, no downgrade. Some imprecision due to moderate sample size, no a priori plan available. Downgrade one level jointly for imprecision and for publication bias  Reason to upgrade not evident  **Rating: Low** |
|  | Collins *et al,* 2003 | 39 | RCT | Some concerns |  | Directly applicable but population specific: subjects with moderate to severe COPD  Visual feedback used and audible tones |  | No a priori plan available  No obvious funding issues or conflict of interest. Research supported by the Department of Veterans Affairs, Veterans Health Administration, Rehabilitation Research and Development Service |  |
| Forced expiratory volume in 1 second/forced vital capacity (FEV_1_/FVC)  2 (59) | Van Gestel *et al*, 2012 | 40 | RCT | High | There is high methodological heterogeneity within these studies  Differences are associated with study design, and protocols, though outcome measured similarly | Directly applicable but population specific: subjects with stable COPD  Visual feedback and acoustic tones used | Imprecision moderate to significant due to moderate to low sample sizes | Registered with ClinicalTrials,gov identifier: NCT01175265. Submission dates suggest an a priori plan not registered  No obvious funding issues or conflict of interest | 2 RCT’s start high:  High risk of bias identified in both studies. Downgrade one level.  Although outcome is the same, there is high inconsistency between studies and conflicting results. Downgrade one level for inconsistency. Both studies directly address the research question, no downgrade for directness, imprecision moderate to significant due to low sample sizes. Publication bias may exist in the older pilot study and likely that an a priori plan not in place for either study. Downgrade one level  **Rating: Very low** |
|  | Esteve *et al,* 1996 | 19 | RCT-pilot study | High |  | Directly applicable but population specific: subjects with stable COPD  Visual feedback used |  | Publication bias may be high in this older pilot study  No obvious funding issues or conflict of interest |  |
| End Tidal PCO_2_  1 (20) | Van Doorn *et al,* 1982 | 20 | RCT | High | Not applicable-single study | Directly applicable but population specific: subjects with chronic hyperventilation syndrome  Visual feedback used | Imprecision significant due to low sample size | No a priori plan available  Study published in 1982  No obvious funding issues or conflict of interest. Research supported by the Netherlands Asthma Foundation | 1 RCT, start high  High risk of bias identified: downgrade one level. Directly applicable to research question. Significant imprecision due to sample size, downgrade one level. No prior plan available, study published in 1982, significant publication bias cannot be ruled out. Downgrade one level  No reason to upgrade evident.  **Rating: Very low** |
| Quality of life (QOL): Perceived breathlessness  3 (128) | van Gestel *et al*, 2012 | 40 | RCT | High | Different measures of perceived breathlessness used. Two studies used CRDQ and one study used BORG scale  Some inconsistency in the reporting of results identified in one study | Directly applicable but population specific: subjects with stable COPD  Subjective nature of outcome measurement a concern  Visual feedback and acoustic tones used | Imprecision moderate due to moderate sample sizes | Registered with ClinicalTrials,gov identifier: NCT01175265. Submission dates suggest an a priori plan not registered  No obvious funding issues or conflict of interest | 3 RCT’s Start high:  Down grade by one level for risk of bias as high for one and some concerns for two. Both measures of breathlessness are relevant. Some inconsistency in outcomes noted with one study reporting inconsistent results in table and text. Downgrade one level. All studies directly relevant to research question, therefore no downgrade for directness. Some imprecision noted and no priori plans available for two studies, therefore strong possibility of publication bias, downgrade one level  **Rating: Very low** |
|  | Collins *et al,* 2008 | 49 | RCT | Some concerns |  | Directly applicable but population specific: subjects with moderate to severe COPD  Subjective nature of outcome measurement a concern  Visual feedback used |  | Detailed on-line supplement available and pre study registration with [www.clinicaltrials.gov](http://www.clinicaltrials.gov) (NCT 00037973)  No obvious funding issues or conflict of interest. Research supported by the Department of Veteran Affairs, Rehabilitation Research and Development, Advanced Career Development Award |  |
|  | Collins *et al,* 2003 | 39 | RCT | Some concerns |  | Directly applicable but population specific: subjects with moderate to severe COPD  Subjective nature of outcome measurement a concern  Visual feedback used and audible tones |  | No a priori plan available  No obvious funding issues or conflict of interest. Research supported by the Department of Veterans Affairs, Veterans Health Administration, Rehabilitation Research and Development Service |  |
| QOL: Perceived fatigue or exertion  3 (128) | van Gestel *et al*, 2012 | 40 | RCT | High | Different measures of perceived fatigue or exhaustion used. Perceived fatigue measured via CRDQ (van Gestel *et al*, 2012: Collins *et al*, 2008). Perceived exertion measured with BORG (Collins *et al*, 2003) | Directly applicable but population specific: subjects with stable COPD  Subjective nature of outcome measurement a concern  Visual feedback and acoustic tones used | Some imprecision due to moderate sample sizes | Registered with ClinicalTrials,gov identifier: NCT01175265. Submission dates suggest a priori plan not registered  No obvious funding issues or conflict of interest | 3 RCT’s Start high:  Down grade by one level for risk of bias as high for one and some concerns for two. Different outcome measures used but both relevant. Some inconsistency in reported outcomes and some imprecision due to low sample numbers. Downgrade one level. Publication bias strongly suspected as no prior plans available for two studies, therefore downgrade one level  **Rating: Very low** |
|  | Collins *et al,* 2008 | 49 | RCT | Some concerns |  | Directly applicable but population specific: subjects with moderate to severe COPD  Subjective nature of outcome measurement a concern  Visual feedback used |  | Detailed on-line supplement available and pre study registration with [www.clinicaltrials.gov](http://www.clinicaltrials.gov) (NCT 00037973)  No obvious funding issues or conflict of interest. Research supported by the Department of Veteran Affairs, Rehabilitation Research and Development, Advanced Career Development Award |  |
|  | Collins *et al,* 2003 | 39 | RCT | Some concerns |  | Directly applicable but population specific: subjects with moderate to severe COPD  Subjective nature of outcome measurement a concern  Visual feedback used and audible tones |  | No a priori plan available.  No obvious funding issues or conflict of interest. Research supported by the Department of Veterans Affairs, Veterans Health Administration, Rehabilitation Research and Development Service |  |
| QOL: Emotion  2 (89) | van Gestel *et al,* 2012 | 40 | RCT | High | Although the same outcome is measured there is some methodological heterogeneity in the studies with conflicting results | Directly applicable but population specific: subjects with stable COPD  Subjective nature of outcome measurement a concern  Visual feedback and acoustic tones used | Some imprecision due to moderate sample sizes | Registered with ClinicalTrials,gov identifier: NCT01175265. Submission dates suggest a prior plan not registered  No obvious funding issues or conflict of interest | 2 RCT’s Start high:  Down grade by one level for risk of bias as high for one and some concerns for two. Although the same outcome is measured there is some methodological heterogeneity in the studies with conflicting results. Down grade one level for inconsistency. Directly applicable to research question, therefore no downgrade for directness. Some imprecision due to low sample sizes and. Likely no a priori plan available for one study therefore down grade one level  **Rating: Very low** |
|  | Collins *et al*, 2008 | 49 | RCT | Some concerns |  | Directly applicable but population specific: subjects with moderate to severe COPD  Subjective nature of outcome measurement a concern  Visual feedback used |  | Detailed on-line supplement available and pre study registration with [www.clinicaltrials.gov](http://www.clinicaltrials.gov) (NCT 00037973)  No obvious funding issues or conflict of interest. Research supported by the Department of Veteran Affairs, Rehabilitation Research and Development, Advanced Career Development Award |  |
| QOL: Mastery  2 (89) | van Gestel *et al,* 2012 | 40 | RCT | High | Although the same outcome is measured there is some methodological heterogeneity in the studies with conflicting results | Directly applicable but population specific: subjects with stable COPD.  Subjective nature of outcome measurement a concern.  Visual feedback and acoustic tones used. | Some imprecision due to moderate sample sizes | Registered with ClinicalTrials,gov identifier: NCT01175265. Submission dates suggest an a priori plan not registered.  No obvious funding issues or conflict of interest. | 2 RCT’s Start high:  Down grade by one level for risk of bias as high for one and some concerns for the other. Although the same outcome is measured there is some methodological heterogeneity in the studies with conflicting results. Down grade one level for inconsistency. Directly applicable to research question, therefore no downgrade for directness. Some imprecision due to low sample sizes and. Likely no a priori plan available for one study therefore down grade one level  **Rating: Very low** |
|  | Collins *et al*, 2008 | 49 | RCT | Some concerns |  | Directly applicable but population specific: subjects with moderate to severe COPD  Subjective nature of outcome measurement a concern  Visual feedback used |  | Detailed on-line supplement available and pre study registration with [www.clinicaltrials.gov](http://www.clinicaltrials.gov) (NCT 00037973)  No obvious funding issues or conflict of interest. Research supported by the Department of Veteran Affairs, Rehabilitation Research and Development, Advanced Career Development Award |  |
| Exercise duration  3 (128) | van Gestel *et al,* 2012 | 40 | RCT | High | Different exercise tests reported. 6MWT (van Gestel et al, 2012), Treadmill (Collins *et al*, 2008) and cycle (Collins *et al*, 2003) | Directly applicable but population specific: subjects with stable COPD  Subjective nature of outcome measurement a concern  Visual feedback and acoustic tones used | Imprecision moderate to significant due to low to moderate sample sizes and limited subject populations | Registered with ClinicalTrials,gov identifier: NCT01175265. Submission dates suggest ana priori plan not registered  No obvious funding issues or conflict of interest. | 3 RCT’s Start high:  Down grade by one level for risk of bias as high for one and some concerns for two. Different exercise tests reported but all relevant. Some inconsistency noted with conflicting results. Downgrade one level. All studies directly relevant to research question, therefore no downgrade for directness. Some imprecision due to low to moderate sample sizes. Publication bias could be significant as no priori plans available for at least two studies  **Rating: Very low** |
|  | Collins *et al,* 2008 | 49 | RCT | Some concerns |  | Directly applicable but population specific: subjects with moderate to severe COPD  Visual feedback used |  | Detailed on-line supplement available and pre study registration with [www.clinicaltrials.gov](http://www.clinicaltrials.gov) (NCT 00037973)  No obvious funding issues or conflict of interest. Research supported by the Department of Veteran Affairs, Rehabilitation Research and Development, Advanced Career Development Award |  |
|  | Collins *et al,* 2003 | 39 | RCT | Some concerns |  | Directly applicable but population specific: subjects with moderate to severe COPD  Visual feedback used and audible tones |  | No a priori plan available.  No obvious funding issues or conflict of interest. Research supported by the Department of Veterans Affairs, Veterans Health Administration, Rehabilitation Research and Development Service |  |
