# Supplementary material for: Biofeedback in Breathing Pattern Training: A Systematic Review
Source: Can Respir J. 2026 Nov 15;2026:5567144. doi: 10.1155/carj/5567144 (PMC13770288; doi:10.1155/carj/5567144)
Supplement: Supplementary file 7 [file CARJ-2026-5567144-s007.docx]

**Supplementary Material 1. PICO framework**

| Population^1^ | Subjects engaging in breathing pattern training |
| --- | --- |
| Intervention^2^ | The use of biofeedback in breathing pattern training |
| Comparator | Usual care or breathing pattern training without the use of biofeedback |
| Outcome | Improved breathing patterns, respiratory symptoms, respiratory outcomes, quality of life measures or functional performance indicators |

^1^Paediatric and adult populations were included in screening. Participants ≥18 years of age were considered as adults. Studies on non-humans or on babies/neonates were not included. Studies investigating the use of BPT during radiological assessment or intervention were excluded. This included BPT within the context of reducing breathing induced organ motion during thoracic imaging and radiotherapy.

^2^There was no restriction on the type of biofeedback used, for example visual, auditory, haptic, or other types of biofeedback were all considered.
