# Supplementary material for: Biofeedback in Breathing Pattern Training: A Systematic Review
Source: Can Respir J. 2026 Nov 15;2026:5567144. doi: 10.1155/carj/5567144 (PMC13770288; doi:10.1155/carj/5567144)
Supplement: Supplementary file 8 [file CARJ-2026-5567144-s008.docx]

**Supplementary Material 4: Inter-rater reliability**

A random number generator was used to identify a small number of studies for inclusion ([www.random.org](http://www.random.org)). Forty studies (5% of 794) were identified.

KB and JM independently and blindly read study titles and abstracts and determined if they would be included for review or not by a binary yes or no. Results were analysed using Kappa co-efficient statistics using IBM SPSS statistics (version 29.0).

Observed agreement – Chance agreement	P_o_ – P_e_

K=

K=

1 – Chance agreement	1 – P_e_
